# Transcriptional profiling of contrasting genotypes revealed key candidates and nucleotide variations for drought dissection in *Camellia sinensis* (L.) O. Kuntze

**DOI:** 10.1038/s41598-019-43925-w

**Published:** 2019-05-16

**Authors:** Rajni Parmar, Romit Seth, Pradeep Singh, Gopal Singh, Sanjay Kumar, Ram Kumar Sharma

**Affiliations:** 1 Biotechnology Department, CSIR-Institute of Himalayan Bioresource Technology (CSIR-IHBT), Palampur, Himachal Pradesh 176061 India; 2 Academy of Scientific and Innovative Research (AcSIR), CSIR-IHBT, Palampur, Himachal Pradesh 176061 India; 3 Department of Biotechnology, Guru Nanak Dev University, Amritsar, 143005 India

**Keywords:** Transcriptomics, Drought

## Abstract

Tea is popular health beverage consumed by millions of people worldwide. Drought is among the acute abiotic stress severely affecting tea cultivation, globally. In current study, transcriptome sequencing of four diverse tea genotypes with inherent contrasting genetic response to drought (tolerant & sensitive) generated more than 140 million reads. *De novo* and reference-based assembly and functional annotation of 67,093 transcripts with multifarious public protein databases yielded 54,484 (78.2%) transcripts with significant enrichment of GO and KEGG drought responsive pathways in tolerant genotypes. Comparative DGE and qRT analysis revealed key role of ABA dependent & independent pathways, potassium & ABC membrane transporters (*At*ABCG22, *At*ABCG11, *At*ABCC5 & *At*ABCC4) and antioxidant defence system against oxidative stress in tolerant genotypes, while seems to be failed in sensitive genotypes. Additionally, highly expressed UPL3HECT E3 ligases and RING E3 ligases possibly enhance drought tolerance by actively regulating functional modification of stress related genes. Further, ascertainment of, 80803 high quality putative SNPs with functional validation of key non-synonymous SNPs suggested their implications for developing high-throughput genotyping platform in tea. Futuristically, functionally relevant genomic resources can be potentially utilized for gene discovery, genetic engineering and marker-assisted genetic improvement for better yield and quality in tea under drought conditions.

## Introduction

Drought is an environmental condition which negatively affects growth, yield and considered as primary cause of crop loss worldwide^1^. Countries like India are facing consecutive drought conditions since last decades and causing significant losses of farm economy and worsening the food security. Tea [*Camellia sinensis* (L.) O. Kuntze], a perennial evergreen woody plant belongs to family Theaceae is highly cross pollinated and heterozygous plantation crop^2^. It is consumed by millions of people worldwide due to its numerous health benefits, wherein, India contribute significantly by being the second largest producer of tea in the world^3^. Nevertheless, tea cultivation is significantly constrained due to the impact of climate change and prolonged rainless periods leading to drought conditions, and reducing tea production by 14–33% with mortality of up to 6–19%^4^. Therefore, tremendous focus of breeders is now on identification and development of high yielding quality tea varieties better adapted to drought conditions^5^. In general, plants are adapted to overcome drought stress *via* avoidance, escape, tolerance and recovery, *vis-à-vis* involvement of several regulatory and functional genes, metabolic and photosynthesis-associated pathways^6,7^.

Drought-induced transitions have been studied earlier at biochemical, physiological and molecular level^8–12^. Biochemical and physiological parameters revealed high water status and photosynthesis rate are key factors involved in osmotic adjustments in tolerant genotypes^8^. Furthermore, transcriptomic studies indicated the putative role of ABA, ethylene and jasmonic acid biosynthesis and signaling, protein kinases and TFs in drought tolerance and recovery^9–12^. Nonetheless, being a multi-genic complex trait, wide range of additional genes are expected to be involved in drought tolerance, wherein, recently reported draft genome sequence information of tea (CSA) might provide better opportunities to elucidate drought stress mechanisms in tea^13^. Considering enormous gene pool with abundance of vigorous and high level of genetic diversity, traditional tea cultivars in India exhibits inherent genetic variations among wide range of desirable traits (quality & yield) including tolerance to drought stress^14^. However, due to out-breeding and long gestation periods, tea require next generation breeding strategies to improve drought tolerance *via* deeper understanding of key regulators and their variants for precision introgressions to have better yield and quality under drought conditions. Therefore, efforts are needed to elucidate global transcriptomic dynamics of multiple tea genotypes having contrasting response to drought stress (tolerant & sensitive) to critically discern key molecular players, which were partly discussed in previous studies in tea.

Current study deals with comprehensive transcriptome sequencing of four tea genotypes exhibiting tolerant (TV 17&TRI2024) and sensitive (TV 03&C 6017) response to drought stress. *de novo* analysis and functional annotations with several databases identified array of genes, regulators and pathways involved in drought stress. In addition to previous study, enrichment analysis of DEGs elucidated the role of novel regulators and genes involved in ABA biosynthesis and transport, antioxidant enzymes, secondary metabolites against oxidative stresses, membrane transporters, ions & osmolytes providing adaptation/tolerance under drought stress in tolerant genotypes. Furthermore, ubiquitin-based regulation of stress hormone signaling and non-proteolytic function during drought stress commencing drought tolerance is also discussed. Additionally, ascertainment and polymorphic potential evaluation of trait specific high quality single nucleotide variations suggest their futuristic implications to expedite the molecular breeding efforts for combining drought tolerance in high yielding quality genotypes of tea.

## Results

### Transcriptome sequencing and assembly

**Figure 1:**
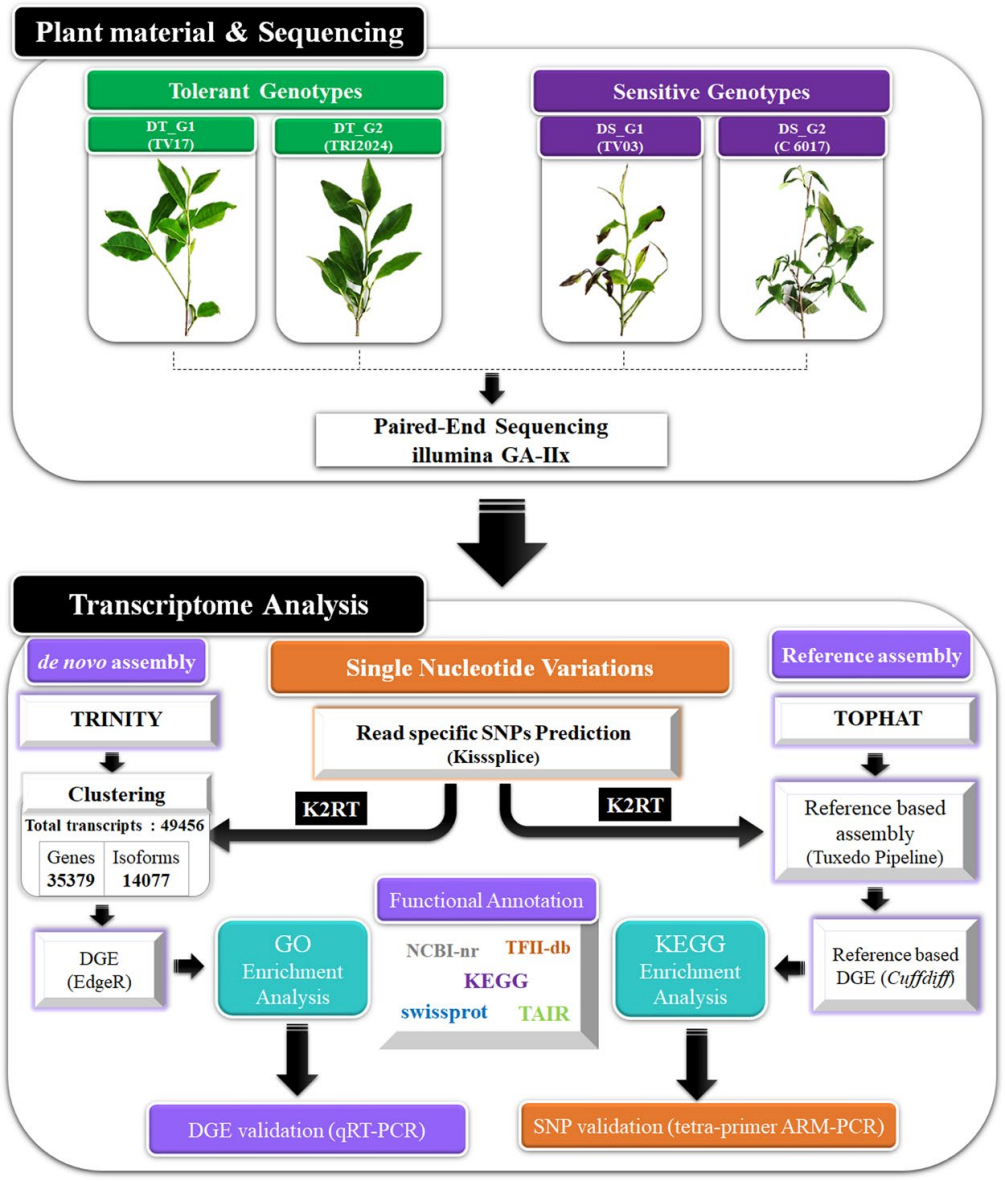
Workflow used in study to dissect drought tolerance in tea.

To gain deeper understanding of global gene(s) dynamics, comprehensive transcriptome sequencing of four traditional tea genotypes, two each of exhibiting inherent tolerant [TV 17 (DT_G1) & TRI 2024 (DT_G2)] & sensitive [TV 03 (DS_G1) & C 6017 (DS_G2)] genetic response to drought stress were sequenced using Illumina Genome Analyser-IIx (Fig. 1). Quality filtering of 140.2 million raw reads with NGS QC Tool kit generated 123.6 million high quality reads. *de novo* assembly using Trinity yielded 67,093 non-redundant (NR) transcripts with average length of 1,086 bp and N_50_ of 1501 bp. Further, CD hit clustering of transcripts with 90% sequence similarity retrieved 54,508 NR transcripts (Supplementary Table S1). Reference-based mapping of high quality reads to CSA genome^13^ detected overall mapping rate of 74.45% to 1,73,311 contigs of draft genome. The raw reads of tea samples were submitted to National Centre for Biotechnology Information (NCBI) Sequence Read Archive (SRA) under the bioproject PRJNA450985 and PRJNA520786.

### Functional annotation and classification

Sequence alignments of 67,093NR transcripts with multifarious public protein databases annotated 55,367 (82.5%) transcripts with NCBI’s Nr (52,484; 78.20%), TAIR (47,962; 71.40%), GO annotations (45,080; 66.3%), Swiss-Prot (41,475; 61.08%), TF database (27,204; 40.54%) and KEGG database (13,454; 20.05%). Meanwhile, transcripts in GO annotation were categorised into Cellular Component (28%), Biological Processes (50%) and Molecular Functions (22%) (Supplementary Fig. S1a). Comparison to KEGG database revealed Enzymes (ko: 01000; 1631 genes), Peptidases (ko: 01002; 120 genes) from metabolism category, Chromosome (ko: 030362; 76 genes), Membrane trafficking (ko: 04131; 239 genes), Spliceosome (ko: 03041; 197 genes) and Ubiquitin system (ko: 04121; 196 genes) from genetic information processing category, and Exosome (ko: 04147; 168 genes) and GTP-binding proteins (ko: 04031; 24 genes) from signaling and cellular processes were among the significantly represented pathways (Supplementary Fig. S1b). Considering, key regulators of plant response against drought stress, 27,204 transcripts encoding 58 TF families with significant abundance of bHLH (2802), NAC (1919), MYB-related (1840), ERF (1581), C2H2 (1289), WRKY (1187), C3H (1137), B3 (1086), FAR1 (995), MYB (958) and bZIP (932) were successfully annotated^9^ (Supplementary Fig. S1c; Table S3).

### Differential gene expression (DEGs) analysis

High quality reads were mapped to *de novo* and reference-genome assembled transcripts to quantify drought responsive differential gene expressions (DEGs)^13^. *De novo* DGEs analysis using control for each treatment in tolerant and sensitive genotypes identified 7473 (DT_G1_C vs DT_G1_T), 4876 (DT_G2_C vs DT_G2_T), 5392 (DS_G1_C vs DS_G1_T), 3220 (DS_G2_C vs DS_G2_T) differentially expressed transcripts. Further, comparison of DEGs in tolerant and sensitive genotypes resulted into 6750 (DS_G1_T vs DT_G1_T), 6997 (DS_G2_T vs DT_G1_T), 6637 (DT_G2_T vs DS_G1_T) and 6766 (DS_G2_T vs DT_G2_T) transcripts. Overall, 3361 and 2250 transcripts were commonly up-regulated in tolerant and sensitive genotypes, respectively. Of these, uniquely up-regulated transcripts were 1849 (DT_G1_T), 1563 (DT_G2_T), 2186 (DS_G1_T) and 2313 (DS_G2_T) (Supplementary Table S4). Whereas, reference genome-based pair-wise comparative DGE analysis resulted in 2056 (DS_G1_T vs DT_G1_T), 3634 (DS_G2_T vs DT_G1_T), 1807 (DT_G2_T vs DS_G1_T), 3267 (DS_G2_T vs DT_G2_T) differentially expressed transcripts (Supplementary Table S5). The significantly less DEGs in reference based analysis possibly due to incomplete & incorrect annotations and exon level difference in expression as also reported in *Arabidopsis*^15^. Therefore, DEGs obtained in *de novo* assembly were utilized in downstream analysis. Expression based clustering to identify key transcripts with similar drought response expression pattern resulted into 5 Sub-clusters (Sub-cluster I: 74 transcripts, Sub-cluster II: 152 transcripts, Sub-cluster III: 87 transcripts, Sub-cluster IV: 74 transcripts and Sub-cluster V: 119 transcripts). Among these, sub-cluster II and III were highly up-regulated in tolerant genotypes, wherein, transcripts encoding E3 ubiquitin ligase involved in plant growth *via* ubiquitination dependant regulation of protein stability were abundant, significantly. Additionally, up-regulated transcription factors (NAC domain-containing protein 100, bZIP11 and WRKY4), ABA transporters (Protein NRT1/PTR family), Glutathione S-Transferase, Chaperone Protein dnaJ, Laccase-15, LEA protein Dc3 and other stress responsive genes possibly promotes drought tolerance *via* ABA mediated signalling, ROS scavenging and maintaining proteins functional conformation in tolerant genotypes^16,17^. Sub-cluster I represents up-regulated transcripts encoding Thaumatin like proteins, Chla-b binding protein, Histone proteins, Phospholipases D beta and Magnesium-chelatase subunit ChlH in DT_G2. Nevertheless, presence of up-regulated expression of few drought responsive genes (14-3-3-like proteins, senescence/dehydration-associated proteins) in sub-cluster IV and V possibly improves stress tolerance under minor drought stress in sensitive genotypes (Supplementary Fig. S3b).

### GO and KEGG enrichment analysis

GO and KEGG enrichment analysis was carried out to critically discern the drought responsive key pathways enriched in tolerant and sensitive genotypes. Among the enriched cellular processes, CUL 4 RING ubiquitin ligase complex (GO: 0009911) and mitochondrial inner membrane (GO: 0005743) were enriched in tolerant and sensitive genotypes, respectively. Nevertheless, intracellular organelle (GO: 0043229) and chloroplast thylakoid membrane (GO: 0009535) were enriched irrespective to tolerant and sensitive genotypes. In biological processes, developmental processes (GO: 0032502), response to stimulus (GO: 0009507, GO: 0048583), regulation of ABA mediated signaling pathway (GO: 0005575) and response to salt (GO: 0003674) were highly enriched in tolerant genotypes. Surprisingly, cellular metabolic processes (GO: 00044237) and cellular amino acid biosynthetic processes (GO: 0009733) were only enriched in drought tolerant quality genotype (DT_G1_T). Moreover, ATP binding (GO: 0005524), calcium-transporting ATPase (GO: 0005388) *via* hydrolase activity (GO: 0004091), ubiquitin-protein ligase activity (GO: 0016567) *via* ligase activity (GO: 0019786), protein kinase activity (GO: 0016740) and oxido-reductase activity (GO: 000018) were highly enriched molecular functions in case of tolerant genotypes. Whereas, cation trans-membrane transporter activity (GO: 0209) and structural constituent of ribosome (GO: 0003735) were the prominently enriched molecular functions in sensitive genotypes (Supplementary Figs S4–S7). Furthermore, tree map representation of KEGG pathways revealed maximal mapping of transcripts in genetic information processing (2328 transcripts), followed by metabolism (2188 transcripts) and signaling & cellular processes (435 transcripts; Supplementary Fig. S1b). Additionally, higher enrichment of flavonoid pathway, amino acid bio-synthesis and porphyrin & chlorophyll metabolism in tolerant genotypes suggests their key relevance to integration of drought stress and quality related traits in tea (Supplementary Fig. S8–S10).

### Dynamics of key pathways involved in drought tolerance

**Figure 2:**
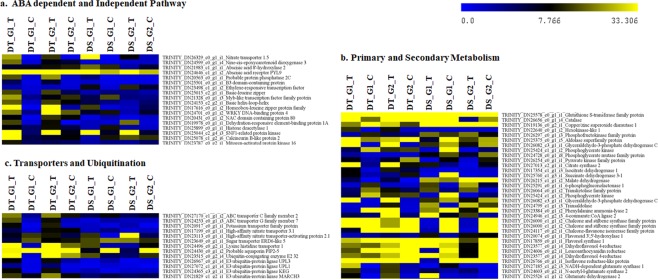
Heat map of differentially expressed genes during drought stress response in tea. (**a**) ABA-dependent and independent pathway (**b**) Primary, Secondary metabolic pathways and antioxidant enzymes (**c**) Transporters and Ubiquitination.

Comprehensive transcriptome analysis of tolerant and sensitive genotypes indicated the role of key pathways such as ABA-dependent and independent pathway, metabolic pathway & antioxidant defense enzymes, membrane transporters and ubiquitinization are crucial for drought tolerance in tea (Fig. 2).

#### ABA-dependent and independent pathway

**Figure 3:**
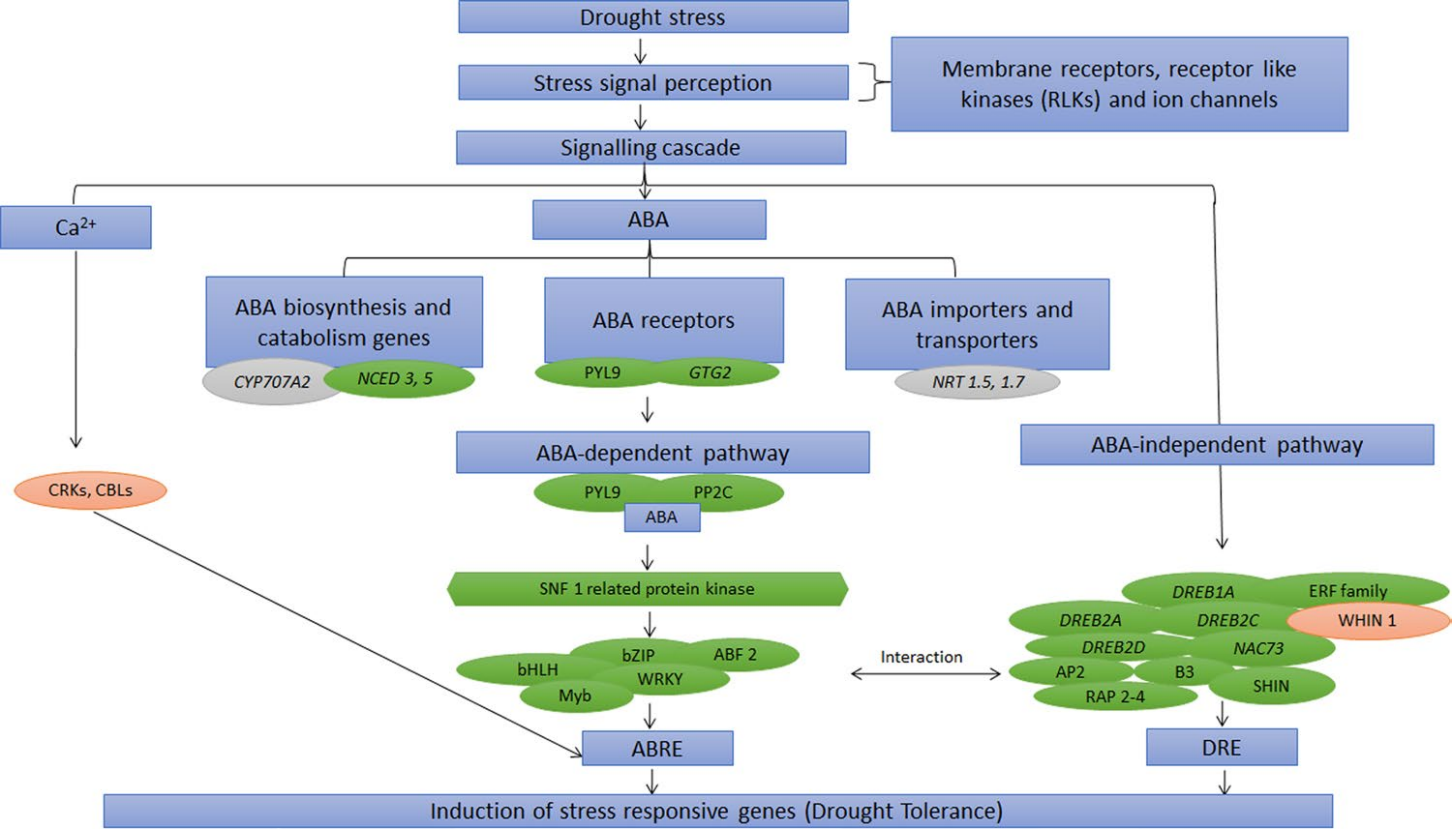
Schematic representation of ABA-dependent and independent signalling during drought response in tea. Boxes in green represent transcripts up-regulated in tolerant while red represents transcripts up-regulated in sensitive genotypes.

ABA regulates various abiotic stress responses in plants, therefore genes involved in ABA metabolism and their specific transporters, receptors and signalling intermediates were scrutinized during the study. Generally, ABA biosynthesis starts in plastids *via* MEP pathway under regulatory control of Zeaxanthin epoxidase (ZEP) and 9-cis-epoxycarotenoid dioxygenase (NCED 5) along with Abscisic acid 8′-hydroxylase 2 (CYP707A2) involved in oxidative catabolism of ABA, interestingly these regulatory elements were up-regulated in tolerant genotypes. Furthermore, import of synthesized ABA to targeted organs for its perception *via* receptor complex to initiate downstream signalling cascade are crucial for activation of drought responsive genes. Up-regulated expression of ABA importers [NRT1/PTR FAMILY (NPF1.2)], ABA transporter (low affinity nitrate transporter) and ABA receptor [GPCR-type G protein 2 (plasma membrane perception) and PYL9 (intracellular perception)] in tolerant genotypes, suggests their precise role during drought stress tolerance^18^. Moreover, ABA-activated protein kinases and phosphatases, having integral involvement in ABA-receptor complex [SNF1-related protein kinase (KING1), Mitogen-activated protein kinase 3 (MPK3), Mitogen-activated protein kinase kinase kinase 1& 7 (MEKKK 1 & 7) and Protein phosphatase 2 C (PP2C)] were also up-regulated in tolerant genotypes. This receptor complex downstream activates ABA-dependent transcription factors such as, bZIP, ABSCISIC ACID-INSENSITIVE 5 (ABF2), Homeobox-leucine zipper protein (ATHB21), bHLH and WRKY TFs regulating drought stress-responsive genes, were also up-regulated in tolerant genotypes. Alternatively, regulation of various drought stress inducible genes in ABA independent manner including dehydration-responsive element-binding protein (DREB1A, 2 A, 2 C & 2D), NAC80, 100 and B3 domain-containing protein were also highly up-regulated in tolerant genotypes. Additionally, up-regulated expression of few histone modifying enzymes (HD1 and HDA15) in tolerant genotypes also indicate their epigenetic impact on stress-responsive genes (Fig. 3; Supplementary Table S6b).

#### Metabolic pathways and antioxidant defence

**Figure 4:**
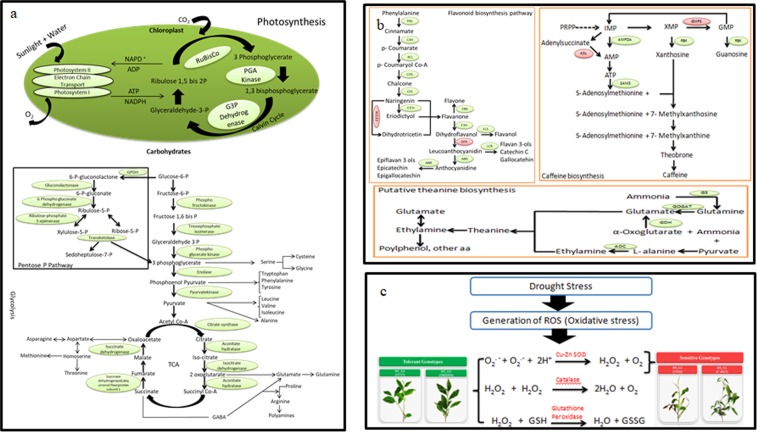
Schematic representation of Primary, Secondary and Antioxidant defence signalling during drought response in tea. (**a**) Primary metabolic pathways (Photosynthesis, Glycolysis and TCA cycle); (**b**) Secondary metabolic pathways (Flavonoid, Theanine and Caffeine biosynthetic pathway); (**c**) ROS detoxification. Boxes in green represent transcripts up-regulated in tolerant while red represents transcripts up-regulated in sensitive genotypes. (PAL: Phenylalanine ammonia lyase, C4H: Cinnamate 4-hydroxylase, 4CL: 4-coumarate CoA ligase, CHS: Chalcone synthase, CHI: Chalcone isomerase, F3′H: Flavonoid 3′-hydroxylase, FNS: Flavone synthase II, F3′5′H: Flavonoid 3′,5′-hydroxylase, F3H: Flavanone 3-hydroxylase, FLS: Flavonol synthase, DFR: Dihydroxyflavonol 4-reductase, LCR: Leucoanthocyanidin reductase, ANS: Anthocyanidin synthase, ANR: Anthocyanidin reductase, GS: Glutamine synthetase, GOGAT: Glutamate synthase, GDH: Glutamate dehydrogenase, ADC: Arginine decarboxylase, GMPS: GMP Synthase, RBK: Ribokinase, ASL: Adenylosuccinatelyase, AMPDA: AMP deaminase, SAMS: S adenosylmethionine synthase).

Most of genes involved in primary (Glycolysis, Citrate cycle, Pentose Phosphate Pathway, Calvin Cycle and Photosynthesis) and secondary (Flavonoid, Theanine and Caffeine) metabolic pathways were up-regulated in tolerant genotypes (Fig. 4a; Supplementary Table S6b). Further, expression analysis revealed that genes involved in flavonoid pathway (PAL2, C4H, 4CLL2, CSH, CHS2, CFI, LAR, ANR, Anthocyanidin reductase and Isoflavone reductase); theanine biosynthetic pathway (GLT, GS-NADH, GDH and ADC2) and caffeine biosynthesis pathway (AMPDA and SAM1) were also found up-regulated in tolerant genotypes (Fig. 4b). Additionally, higher KEGG enrichment of flavonoid, amino acid biosynthesis and porphyrin & chloroplast metabolism in tolerant genotypes also complements the expression data (Supplementary Fig. S8–S10; Table S6).

Prolonged and severe drought conditions undoubtedly lead to oxidative stress which affects the primary metabolic activity of the cell. Up-regulated expression of superoxide dismutase irrespective to tolerant and sensitive genotypes suggest its involvement to maintain first line of defence in all genotypes, while downstream H_2_O_2_ scavenging enzymes, namely Glutathione S transferase, Catalase and Ascorbate peroxidase only expressed in tolerant genotypes were possibly involved to cope-up with drought stress in tolerant genotypes (Fig. 4c).

#### Membrane transport dynamics during stress adaptation

Despite important role in stress adaptation, plant membrane transport system was least discussed in tea. During this study, efforts were made to critically understand the role of various transporter families such as ABC, Ion transporters and Aquaporin channels involved in osmotic adjustments during drought stress (Supplementary Table S6b).

**Figure 5:**
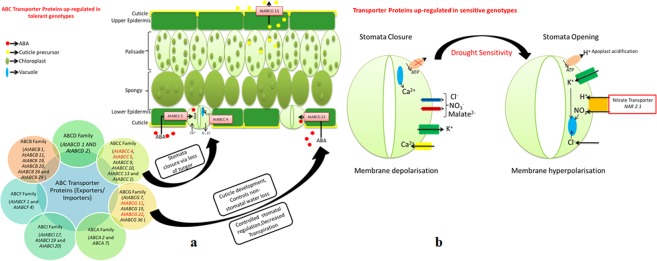
Illustration showing role of transporters in opening and closing of stomata during drought stress response in tea. (**a**) ABC transporter activity in tolerant genotypes. (**b**) Nitrate transporter activity in sensitive genotypes.

ABC Transporters: Expression data revealed active involvement of ABC protein superfamily during drought stress in tolerant and sensitive genotypes. Among these, members of ABCG subfamily namely *At*ABCG 1 (ABC transporter G family member 1, 7, 11, 15, 22 & 36) reportedly involved in stomatal function and ABCC subfamily (*At*ABC C4, 5, 9, 10 & 13) participates in the guard cell physiology/stomatal regulation and root development, exhibited higher expression in tolerant genotypes (Fig. 5a). Moreover, ABCB transporter proteins (*At*ABCB 1, 11, 19, 20, 28 & 29) involved in auxin mediated development and transport; and few additional transporters with unknown functions such as ABC Transporter I (*At*ABCI 17, 19 & 20), ABC Transporter D (*At*ABCD 1 & *At*ABCD 2), ABC Transporter A (ABCA 2 & ABCA 7) and ABC Transporter F (*At*ABCF 1 & *At*ABCF 4) were also highly up-regulated in tolerant genotypes.

Ion Transporters: Ion transporters not only help to maintain sustainable plant growth and productivity but also important for immediate drought response *via* rapid and fast adjustment of stomatal aperture to minimize transpirational water loss. Transcripts encoding potassium transporters (POT 1, 2 and HAK 25) and Zinc transporters (ZIP 5 and ZIP 6) were expressed in tolerant genotypes. Contrarily, higher expression of nitrate transporter (NAR2.1) reportedly involved in stomatal opening probably causing excess water loss in sensitive genotypes (Fig. 5b).

Amino acid transporters: Amino acid transporters provide drought tolerance in plants *via* osmotic adjustments to protect cells from dehydration by increasing cellular osmolarity^19^. Higher expression of Lysine histidine transporters (LHT1 and AATL1) in tolerant genotypes further support their involvement in controlling osmolarity to acquire tolerance during drought stress.

Aquaporins: Aquaporins are membrane channel proteins having role in plant growth, development and defense against drought stress^20^. Expression of transcripts encoding three major subfamilies *viz*; Plasma Membrane Intrinsic Proteins (PIPs) and Tonoplast Intrinsic Proteins (TIPs) of aquaporins revealed significant differential expression among tolerant and sensitive genotypes. Of the two PIP subgroups (PIP1, PIP2; isoform with unique localization and functions), PIP1-2, PIP2-5, PIP2-7 was up-regulated in tolerant genotypes, and among TIPS, TIP1-1, TIP2-1, TIP3-1 and TIP1-3 participates in water exchange were also up-regulated in tolerant genotypes.

#### DGEs of Ubiquitination complex during drought response

Key mechanism underlying functional modification during drought stress is still obscure in tea. Ubiquitination, a protein modification mechanism involved in cellular signalling begins with active participation of three important enzymes to facilitate ubiquitin-mediated degradation of target proteins, different stress responsive TFs and hormonal receptors. Among these, E1 Ubiquitin-activating and E2 Ubiquitin-conjugating enzymes (UBC 2, 5, 7, 16, 24, 25, 27 and 28) were expressed irrespective to tolerant and sensitive genotypes, while, E3s Ubiquitin ligase mainly UPL3 HECT E3 ligases, RING-E3 ligases and CHIP U-box E3 ligase involved in leaf senescence, ABA stress related signalling and protein turnover metabolism, respectively were up-regulated in tolerant genotype. Additionally, Cullin-RING ligases (CRLs) and Skp1-cullin-F-box (SCF) involved in plant hormone assisted drought stress adaptation were up-regulated irrespective to all genotypes, whereas, BTB (bric-a-brac-tramtrack-broad) complex, DNA damage-binding (DDB) and Anaphase-promoting complex (APC) were up-regulated only in tolerant genotypes. Furthermore, ubiquitin like modifiers (UBLs) such as, NEDD8, SUMO-UBC9, SUMO-SIZ1 and UFL1 was up-regulated in tolerant, however MMS1 SUMO conjugating enzyme was more active in sensitive genotypes.

#### Expression of TFs in response to drought stress

Among the ascertained transcription factor (TF) families, transcripts encoding bHLH, NAC, WRKY, B3, MYB and bZIP were upregulated more in tolerant genotypes, while, C2H2 domain up-regulated in sensitive genotypes. Among these, high expression of bHLH 13, 48 and NAC 2, 78 possibly providing drought tolerance *via* activating ABA signalling in tolerant genotypes^21^. Moreover, tolerant genotype specific up-regulated expression MYB family (Myb39, 44) and WRKY (WRKY 4, 19, 24, 33 & 75) might improve drought tolerance *via* stomata closureby increasing osmotic stress response. Additionally, higher expression of bZIP 19, 23 in tolerant genotypes was reported to be crucial in abiotic stress signalling^17^.

### Real Time quantitative PCR expression analysis

**Figure 6:**
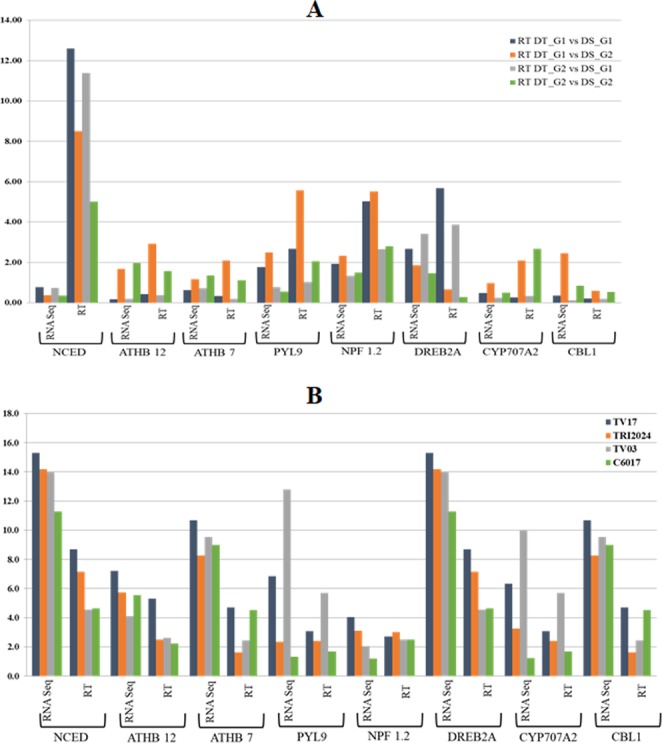
Comparison between RNA-seq and qRT-PCR expression profile of 8 drought related genes in Control vs Treatments and Tolerant vs Sensitive genotypes.

To validate key drought responsive candidates, quantitative real time (qRT-PCR) expression pattern of random eight genes representing major pathways (ABA-dependent and independent pathway and Ubiquitination) were corresponded with RNA seq expression data (Fig. 6; Supplementary Table S7).

### Discovery and validation of drought responsive putative single nucleotide polymorphism

Single nucleotide polymorphism (SNP) ascertained from trait specific transcriptome analysis have been successfully utilized for high-throughput genome mapping and discovery studies^22^. Using stringent pipeline, 80,803 high quality putative SNPs localized in CDS were predicted in 28,096 transcripts by mapping high quality filtered RNA-Seq reads to *de novo* and reference genome of tea. Of these, 28045 SNPs were non-synonymous, while 39679 SNPs categorized as synonymous. Among the non-synonymous SNPs, transversions (A/C: 1060, G/T: 32107, C/G: 1851, T/A: 1326) were more abundant than transitions (A/G: 7395 and C/T: 3224). Interestingly, SNPs ascertained in major drought specific pathways varied from 40 SNPs identified in ABA dependent and independent to 79 SNPs in primary and secondary metabolism, while 61 SNPs and 76 SNPs identified in transporters and ubiquitination encoding transcripts, respectively (Supplementary Fig. S11; Table S8). GO enrichment analyses of SNPs containing transcripts revealed enrichment of several drought responsive GO categories in contrasting genotypes. Among the GO enrichment in tolerant genotypes, biological processes category; regulation of biological processes (GO: 0050789), primary metabolic processes (GO: 0044238), developmental processes (GO: 0032502), response to stimulus (GO: 0050896) and response to abiotic stimulus (GO: 0009628) were highly enriched, while, metabolic processes (GO: 0008152) was the only category moderately enriched in sensitive genotypes. Interestingly, molecular function category, protein binding (GO: 0005517) were only enriched in tolerant genotypes, whereas, cell (GO: 00005623) and cell parts (GO: 0044464) of cellular components were found to be enriched irrespective to tolerant and sensitive genotypes (Supplementary Fig. S12–S14).

### Allele specific functional validation of drought responsive SNPs

In total, 37 non-synonymous SNPs ascertained in key drought responsive pathway genes and localized to CDS region were utilized for amplification validation in 24 tea genotypes (Supplementary Table S9). Of these, 26 putative SNPs were successfully validated using allele specific PCR with four primer combinations wherein two allele specific inner primers were used to discriminate the single nucleotide change between different genotypes (Supplementary Fig. S15). Considering, derivation of validated SNPs in the transcripts encoding drought stress responsive genes such as, RAP2–4, PP2C, RAP2–12, ERF 118, WRKY 118, 4, NAC 17, NPF2.11, NPF 5.2, RAP2–3, ERF 18, MKK9 (ABA dependent and Independent pathway), Glutathione-S transferase (Antioxidant defence), ABCG1, 7, ABCB 9, ABCF 1 (Transporters), F3H, ASS, CS2, ACO, SDHAF4, IDH and RBCL (Primary & Secondary metabolism) and XERICO (Ubiquitination), might be having functional and adaptive significance against drought stress.

## Discussion

### Transcriptome analysis

Being polygenic complex trait, achieving genetic gain with single/few gene(s) under drought stress is difficult. Therefore, it is crucial to comprehensively understand the genetic control of drought tolerance in plants. In current study, high throughput transcriptome sequencing of multiple traditional genotypes with contrasting inherent genetic response to drought stress was performed to provide better perspective of the molecular machinery operating in drought stress in tea^23^. Combined approach using both reference-based and *de novo* transcriptome analysis provides better resolution of key candidates underlying in drought stress associated metabolic network^24^. Overall, high transcriptome read mapping (74.45%) obtained with CSA genome suggests high quality assembly of short reads in this study. Nevertheless, metrics used in genome assemblies are not suitable for transcriptome assembly due to abundance of chimerism in longer sequences, hence *de novo* assembly is preferred in global transcriptional analysis^15^. Therefore, considering multiple advantages including better opportunity of abundance of novel candidates, TRINITY assisted *denovo* assembly was utilized for downstream dissection of drought tolerance in the current study^23,25^.

Approximately 123 million high quality reads (67,093 transcripts with an average length: 1086 bp; N50: 1501 bp) were obtained here are comparable to the earlier studies in tea and other complex crops^10,26^. Overall functional annotation of 78.2% transcripts suggests that optimal annotation of the transcriptome data. Moreover, higher GO enrichment of important relevant biological processes; response to stimulus and regulation of abscisic acid mediated signaling pathway indicates activation of drought tolerance responsive genes in tolerant genotypes. Although, overall DEGs was found higher in sensitive as compare to tolerant genotypes, yet, key transcripts involved in drought tolerance were highly up-regulated in tolerant genotypes^27–29^.

### Signal transduction *via* ABA-dependent and Independent Pathways

ABA plays critical role in drought stress and seems to work efficiently in tolerant genotypes. Drought stress signal is sensed by membrane receptors which downstream activate signalling cascade to generate secondary signal molecules viz., Ca^2+^, ROS and ABA. Higher expression of ABA biosynthesis and catabolism enzymes in tolerant genotypes indicates the fine tuning of synthesis and degradation of ABA during drought stress in tolerant genotypes^30^. Furthermore, both synthesis and transport of ABA to site of action is crucial for stress response and can be correlated extensively with stomatal closing. Up-regulation of NRT1/PTR FAMILY members possibly stipulate their role in stomatal closure under drought stress to minimize water loss in tolerant genotypes^16^. While, perception of ABA to regulate downstream expression of various ABA-responsive genes is important to promote drought stress tolerance. Higher expression of PYL9 receptor in tolerant genotypes provides putative evidences that these receptors in the presence of ABA following ABA core signalling to form receptor-complex. This complex possibly trigger phosphorylation and activation of SNF1-related protein kinases to phosphorylate bZIP, ABF2, MYB and WRKY transcription factors by regulating ABRE-dependent signalling cascade to improve drought stress in tolerant genotypes. Incase of ABA-independent pathway, dehydration induced DREB proteins and perhaps mediate transcriptional regulation of osmotic stress responsive genes. Higher expression of DREB2A subfamily in tolerant genotypes reveals that DREB subfamily could be one of the putative key candidates, which can be futuristically explored to enhance drought tolerance in tea^18^.

### Detoxifying enzymes and secondary metabolites against oxidative stress

Drought stress enhances production of different oxidative free radicals (O_2_^−^, OH^−^ and H_2_O_2_) consequently causes damage to photosynthetic machinery of cell. Antioxidant defence system is an adaptative strategy, wherein, superoxide dismutase causes dis-mutation of O_2_^۰−^radical to H_2_O_2_ and O_2_ which is further scavenged to water and oxygen by downstream enzymes viz., Catalase and Glutathione peroxidase. Higher expression of SOD in both tolerant and sensitive genotypes; and lower expression of downstream antioxidant defence enzymes in sensitive genotypes reveals that first line of defence is working efficiently irrespective to tolerant and sensitive genotypes however, downstream H_2_O_2_ scavenging enzymes could be the key factor for failure of drought tolerance in sensitive genotypes. Hence, up-regulated expression of SOD with active H_2_O_2_ scavenging enzymes may be a putative mechanism to handle oxidative stress during drought in tea^31^. Moreover, stress-induced higher expression of non-enzymatic antioxidants like flavonoids in tolerant genotypes suggests that active metabolites possibly involved to cope-up with oxidative damage and perhaps helped for maintaining tea quality during drought stress^4^.

### Role of transporters in stress adaptation in tea

ABC transporter proteins, the largest known transporters of ABA and auxin involved in stomatal functioning were highly up-regulated in tolerant genotypes during drought stress. Higher expression of lipid transporter, *At*ABCG 11 indicates that cuticle covering in tolerant genotypes might protects plant from dehydration *via* controlling non-stomatal water loss^32^. Similarly, higher expression *At*ABCG 22 probably involved in stomatal closure *via* enhanced influx of ABA into guard cells of tolerant genotypes. Additionally, up-regulated transporter subfamily *At*ABCC 5 and *At*ABCC 4 suggests that stomata closure due to loss of turgor in guard cells *via* Cl^−^ and K^−^ ion efflux along with ABA accumulation in tolerant genotypes, but not in sensitive genotype hence may also be key molecular players involved in drought adaptation.

Controlled regulation of potassium transporters *via* ABA and auxin is key factor during osmotic adjustments. Higher expression of these genes reflects activation of potassium ion transport system leading to potassium homeostasis in guard cells and regulating stomatal aperture in tolerant genotypes^33^. Accumulation of copper and zinc ions in stressed plants *via* COPT 5, ZIP 5 and ZIP 6 transporters possibly involved in free radical removal and signalling in tolerant genotypes^34,35^. Interestingly, up-regulation of nitrate transporter (NAR2.1) in sensitive genotypes suggests accumulation of nitrate in guard cells inducing depolarization enhances stomatal opening, causing drought sensitivity^36^. Higher expression of LHT transporters and aquaporins stipulates water scarcity in tolerant genotypes is possibly managed *via* increasing cellular osmolarity during drought stress^19,20^.

### Functional modification in response to drought stress

Ubiquitination, key mechanism necessary for various cellular signalling triggers drought stress response. Higher expression of UPL3 HECT E3 ligase might delayed leaf senescence *via* degradation of WRKY53 gene during drought tolerance^37^. Further, up-regulated expression of various RING E3 ligases such RGLG2, XERICO and SDIR1 possibly stipulate drought stress adaptation *via* controlling ABA signalling in tea^38,39^. Additionally, up-regulated expression of RMA1H1 RING E3 ligases regulates aquaporin trafficking to plasma membrane in tolerant genotypes possibly suggests ubiquitin mediated regulation of aquaporin level during drought stress^40^. Nevertheless, up-regulated expression of PUB23 (negative regulator of drought stress) might resulted in dehydration and failing drought tolerance in sensitive genotypes^41^. Higher expression of CRLs (BTB, DDB and APC) perhaps anticipates strong hormonal responses in tolerant genotypes. Further, drought response protein degradation caused due over-expression of KEG E3 ligases is perhaps maintained by up-regulated expression of SIZ1 *via* sumoylation in tolerant genotypes^42^. Thus, expression data reveals that ubiquitination system work effectively *via* complex regulatory hormone responses and downstream signal transduction cascade in tolerant genotypes of tea.

### SNPs in drought stress responsive genes

To increase the resolution of genetic changes involved during drought tolerance, high throughput mining of candidate SNPs in transcriptome data and their utilization for genotyping and genome-wide association studies have been appropriately utilized in trait dissection^43^. Moreover, SNP ascertained from transcriptomic data had a higher success rate than the whole genome resequencing data^44^. Ascertainment of 80803 high quality putative SNPs from multiple genotypes with significant abundance in key drought responsive pathways [ABA dependent and independent (40 SNPs), Antioxidant defense (79 SNPs), transporters (61 SNPs) and ubiquitination (76 SNPs)] suggests higher success rate in marker validation and their implications in drought dissection in tea^22^. Furthermore, unique abundance of non-synonymous functional SNPs (CDS region) can affect the thermostability and activity of gene in contrasting genotypes (tolerant: 1178 SNPs and Sensitive: 334 SNPs) and experimental validation of 37 trait-specific SNPs suggests potential implications of ascertained SNPs in QTLs analysis of drought tolerance in tea^22^.

## Conclusion

Tea quality and yield have been significantly constraint due to negative impact of drought stress. In the current study, global transcriptome analysis using multiple diverse genotypes with inherent contrasting response to drought (Tolerant & Sensitive) followed by *de novo* and reference guided genome assembly and annotation with multiple public databases have comprehensively enriched the drought responsive genomic resource in tea. Comparative transcriptional analysis successfully identified novel regulatory and putative functional candidates providing drought tolerance in tea *via* ABA dependent & independent stress signalling, membrane transporters (*At*ABCG22, *At*ABCG11, *At*ABCC5 & *At*ABCC4) and aquaporins (Figs 3, 5). Moreover, antioxidant defence enzymes against oxidative stress and post-translational modification of various drought responsive genes *via* ubiquitination and sumoylation can be additional potential candidates for enhancing drought tolerance in tea (Fig. 4c). Additionally, abundant genomic resources comprising potential key candidates and trait-specific putative SNPs can be futuristically utilized for combining drought tolerance in high yielding quality tea genotypes using genetic engineering and genome mapping studies.

## Materials and Methods

### Plant material and treatment

Four traditional genotypes of tea [*C*. *Sinensis* (L) O. Kuntze] exhibiting inherent tolerant [TV 17 (DT_G1), TRI 2024 (DT_G2)] and sensitive [TV 03 (DS_G1) and C 6017 (DS_G2)] response to drought stress used in this study were maintained at CSIR- Institute of Himalayan Bio-resource Technology Palampur, Himachal Pradesh (1,300 m Altitude, 32° 06′N, 76° 33′E). Shoot cuttings of tolerant and sensitive plants were collected during actively growing period (April, 2017; IST 10–11am) in de-ionised water and incubated in plant growth chamber at 25 ± 1 °C for 24 hrs. Thereafter, cuttings of tolerant and sensitive genotypes were transferred to half-strength Hoagland’s nutrient medium containing 10% polyethylene glycol 8000 (PEG 8000, Formally 6000, USB) for a period of 48 hours to induce uniform drought stress in relatively controlled manner alongwith control samples for each genotypes^45^. Four leaves and a bud tissues from all four genotypes was harvested after 48 h, immediately frozen in liquid nitrogen and stored at (−) 80 °C for RNA isolation.

### RNA isolation, cDNA library preparation and sequencing

Total RNA isolation was carried out using *iRIS* protocol followed by quantity and quality determination using Nanodrop 2000 (Thermo Scientific Lithuania) and Agilent Bio-analyzer Chip RNA 7500 series II (Agilent Technologies USA)^46^. Equimolar concentration of total RNA from three technical replicates was pooled prior to cDNA library preparation for minimising biological biasness. 4 µg of RNA from each sample was used for construction of cDNA library using illumine TruSeq RNA Sample Prep Kit v2 LS (Illumina Inc., San Diego, CA). Prepared libraries were quantified using Bio-analyser Chip DNA 1000 Series II (Agilent Technologies USA). Diluted libraries of 10 pM concentration were loaded on to the flow cell for paired end (PE) sequencing of (2 × 72 bp) using Illumina Genome Analyser-IIx (Illumina, San Diego, CA).

### *De novo* assembly and functional annotation

Base calling and de-multiplexing of raw data was performed using Illumina Casava 1.8.2 pipeline (http://support.illumina.com/) followed by Paired-End quality filtering using NGS QC Toolkit^47^. Trinity Software package ver2.3.2 was utilized for *de novo* transcriptome assembly with minimum cut-off length of 300 bp, further CD hit clustering tool was used to cluster the transcripts with 90% sequence similarity^48,49^. Homology based putative function prediction of assembled transcripts with various publically available databases through BLASTx similarity search was performed viz., NCBI Nr (Non-Redundant), The Arabidopsis Information Resource (TAIR 10), Swiss-Prot, KEGG automatic annotation server (http://www.kegg.jp/kegg/tool/annotate_sequence.html) and Plant Transcription Factor database (http://planttfdb.cbi.pku.edu.cn/) with an e-value of ≤ 1e −5.

### Differential expression analysis, GO and KEGG pathway enrichment analysis

For identification of differentially expressed (DE) transcripts among tolerant and sensitive genotypes, sample specific filtered reads were mapped to both *de novo* assembled transcripts and reference genome using Bowtie2 ver2.2.4 and Tuxedo pipeline respectively^50,51^. In *de novo* analysis, transcript abundance was estimated and normalised to FPKM (Fragments per Kilobase of Transcript per Million Mapped reads) using RSEM tool ver1.2.31^52^. Differential transcripts expression was performed using edgeR package in drought tolerant control vs drought tolerant treated (DT_G1_C vs DT_G1_T and DT_G2_C vs DT_G2_T), drought sensitive control vs drought sensitive treated (DS_G1_C vs DS_G1_T and DS_G2_C vs DS_G2_T), drought tolerant control vs drought sensitive control (DT_G1 vs DS_G1, DT_G1 vs DS_G2, DT_G2 vs DS_G1 and DT_G2 vs DS_G2) and drought tolerant treated vs drought sensitive treated (DT_G1 vs DS_G1, DT_G1 vs DS_G2, DT_G2 vs DS_G1 and DT_G2 vs DS_G2)^53^. Transcripts with log2 FC >1, p-value ≤ 0.05 were considered for downstream analysis. Further, significant DE transcripts (FC ≥2; p-value < 1e-4) were extracted and clustered based on similar expression pattern. Moreover, reference based DE analysis was performed using TopHat ver2.1.0 to extract significantly DE transcripts with P-value < 0.05. Heat map-based representation of DE transcripts of various pathways was developed using MeV package v.4.9.0 (Multiple Experiment Viewer). Statistically significant DE transcripts were subjected to GO and KEGG enrichment analysis *via* agriGO tool and KEGG pathway database respectively^54,55^. Singular enrichment analysis (SEA) was performed to extract the GO enrichment of differentially expressed transcripts using AgriGO toolkit with fisher’s statistical test (Hochberg-FDR adjustment, Cut off <0.01) for optimal gene enrichment^54^.

### Quantitative Real Time PCR (qRT-PCR) expression analysis

Gene expression inferences obtained in RNA-Seq data were validated using qRT-PCR. DNase I (Thermo Scientific) treatment was given to RNA samples to remove any contamination of DNA. From all four genotypes, 4 µg of RNA were used for cDNA synthesis using cDNA synthesis kit (Thermo Scientific, Revert H Minus) and 10X dilutions were used for qRT-PCR. Gene specific primers were designed using BatchPrimer3 (http://probes.pw.usda.gov/batchprimer3/) (Supplementary Table S7). Differential expression pattern of 8 genes from important pathways induced during drought stress among tolerant and sensitive genotypes were analyzed (StepOnePlus Real-Time PCR system, Applied Bio-system, USA) using 18 s as internal control with three technical replicates to calculate standard error. Equal concentration of cDNA in each reaction was established using internal control and relative expression of genes and fold change was calculated using 2^−ΔΔCT^protocol^56–59^.

### SNPs prediction from *de novo* and reference genome

Read specific single nucleotide variations were ascertained among all four genotypes utilizing KISSPLICE ver 2.4 with default parameters. BLAT *ver*. 3.6 was utilized to extract the positioned variants in both *de novo* and reference genome assembly; and their functional impact were predicted using kISSPLICE2REF TRANSCRIPTOME (K2RT)^60^.

### Tetra Primer ARM PCR for SNPs validation

Tetra primer ARM PCR is fast and inexpensive method for genotyping and also distinguishes homozygote from heterozygote SNPs. In current study, 37 drought stress responsive transcripts containing non-synonymous SNPs were used for allele specific primer designing utilizing online programme PRIMER1: Primer design for tetra-primer ARMS-PCR^61^ (http://primer1.soton.ac.uk/primer1.html). Two outer primers (FOP and ROP) and two allele specific inner primers (FIP and RIP) were designed along with a mismatch base added to the 3′ end of inner primers and fragment size was kept between 100–450 bp. SNPs were validated in 24 diverse genotypes of tea (including four genotypes used for transcriptome study^62^ (Supplementary Table S9). Total genomic DNA was isolated using DNeasy Plant Mini Kit (Qiagen, Germany) according to manufacturer’s instructions. Quantity and quality of DNA was determined using NanoDrop 2000 OD_260_/OD_280_ (Thermo Scientific, Lithuania) and integrity using 0.8% agarose gel. PCR amplification was performed using 40 ng of genomic DNA and amplified PCR products were separated on 3% metaphore agarose gel, visualised on UV trans-illuminator (BioRad).

## Supplementary information

Supplementary Information

Supplementary Table S3

Supplementary Table S4

Supplementary Table S5

Supplementary Table S6a

Supplementary Table S6b

Supplementary Table S7

Supplementary Table S8

Supplementary Table S10
