# Supplementary material for: Transcriptional profiling of contrasting genotypes revealed key candidates and nucleotide variations for drought dissection in Camellia sinensis (L.) O. Kuntze
Source: Sci Rep. 2019 May 16;9:7487. doi: 10.1038/s41598-019-43925-w (PMC6522520; doi:10.1038/s41598-019-43925-w)
Supplement: Supplementary file 1 — Supplementary Information [file 41598_2019_43925_MOESM1_ESM.pdf]

Supplementary Information

Transcriptional profiling of contrasting genotypes revealed key candidates and nucleotide variations for drought dissection in *Camellia sinensis* (L.) O. Kuntze

Rajni Parmar[1,2], Romit Seth[1,3], Pradeep Singh[1,3], Gopal Singh[1,2], Sanjay Kumar[1,2], Ram Kumar Sharma[1,2]*

[1]Biotechnology Department, CSIR-Institute of Himalayan Bioresource Technology (CSIR-IHBT), Palampur, Himachal Pradesh, India

[2]Academy of Scientific and Innovative Research (AcSIR), CSIR-IHBT, Palampur, Himachal Pradesh, India

[3]Department of Biotechnology, Guru Nanak Dev University, Amritsar, 143005, India

*__Correspondence:__
Dr Ram Kumar Sharma
Biotechnology Division
CSIR-Institute of Himalayan Bioresource Technology
Palampur (H.P)
India
Email: rksharma.ihbt@gmail.com, ramsharma@ihbt.res.in

Figure S1

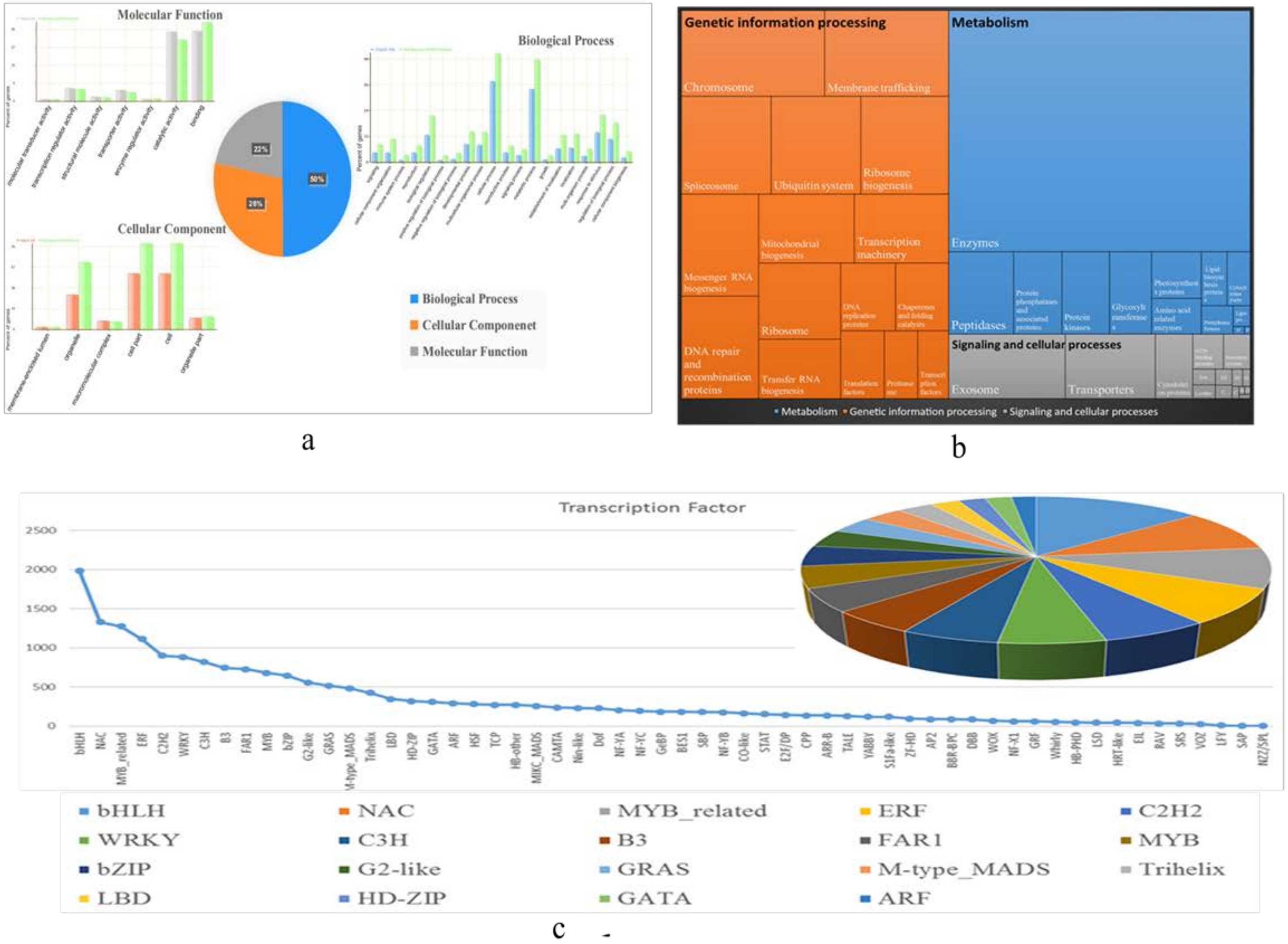

Molecular Function
Biological Process
Cellular Component
50%
28%
22%
Biological Process
Cellular Componenet
Molecular Function
a
Genetic information processing
Metabolism
Chromosome
Membrane trafficking
Spliceosome
Ubiquitin system
Ribosome biogenesis
Mitochondrial biogenesis
Transcription machinery
Messenger RNA biogenesis
Ribosome
DNA repair and recombination proteins
Transfer RNA biogenesis
Enzymes
Peptidases
Protein kinases
Signaling and cellular processes
Exosome
Transporters
Metabolism
Genetic information processing
Signaling and cellular processes
b
Transcription Factor
2500
2000
1500
1000
500
0
bHLH
NAC
MYB_related
ERF
C2H2
WRKY
C3H
B3
FAR1
MYB
bZIP
G2-like
GRAS
M-type_MADS
Trihelix
LBD
HD-ZIP
GATA
ARF
c

Figure S2

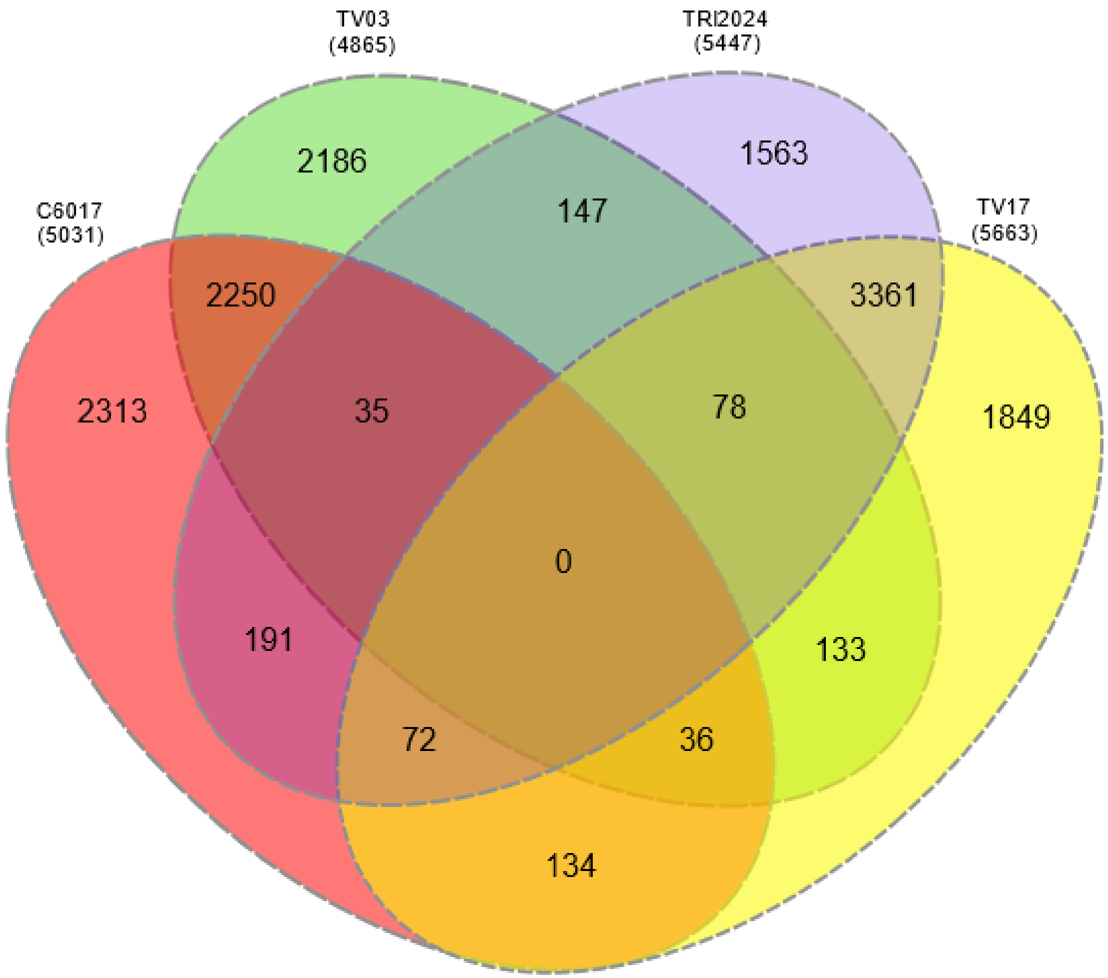
TV03
(4865)
TRI2024
(5447)
C6017
(5031)
TV17
(5663)
2186
1563
147
2250
3361
2313
35
78
1849
0
191
133
72
36
134

Figure S3a

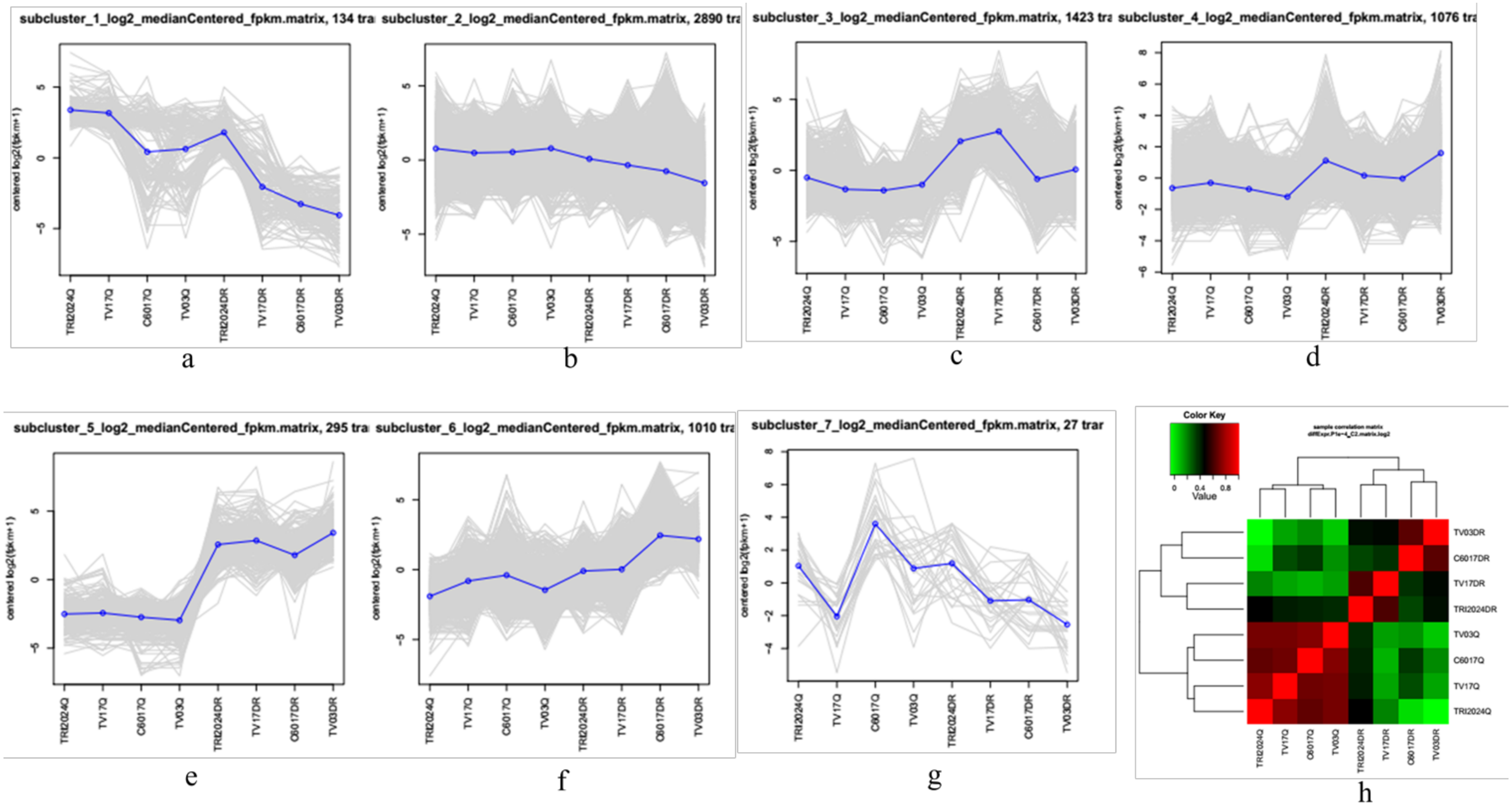
subcluster_1_log2_medianCentered_fpkm.matrix, 134 tra
subcluster_2_log2_medianCentered_fpkm.matrix, 2890 tra
subcluster_3_log2_medianCentered_fpkm.matrix, 1423 tra
subcluster_4_log2_medianCentered_fpkm.matrix, 1076 tra
subcluster_5_log2_medianCentered_fpkm.matrix, 295 tra
subcluster_6_log2_medianCentered_fpkm.matrix, 1010 tra
subcluster_7_log2_medianCentered_fpkm.matrix, 27 trar
centered log2(fpkm+1)
TRI2024Q
TV17Q
C6017Q
TV03Q
TRI2024DR
TV17DR
C6017DR
TV03DR
Color Key
Value
0
0.4
0.8
sample correlation matrix
a
b
c
d
e
f
g
h

Figure S3b

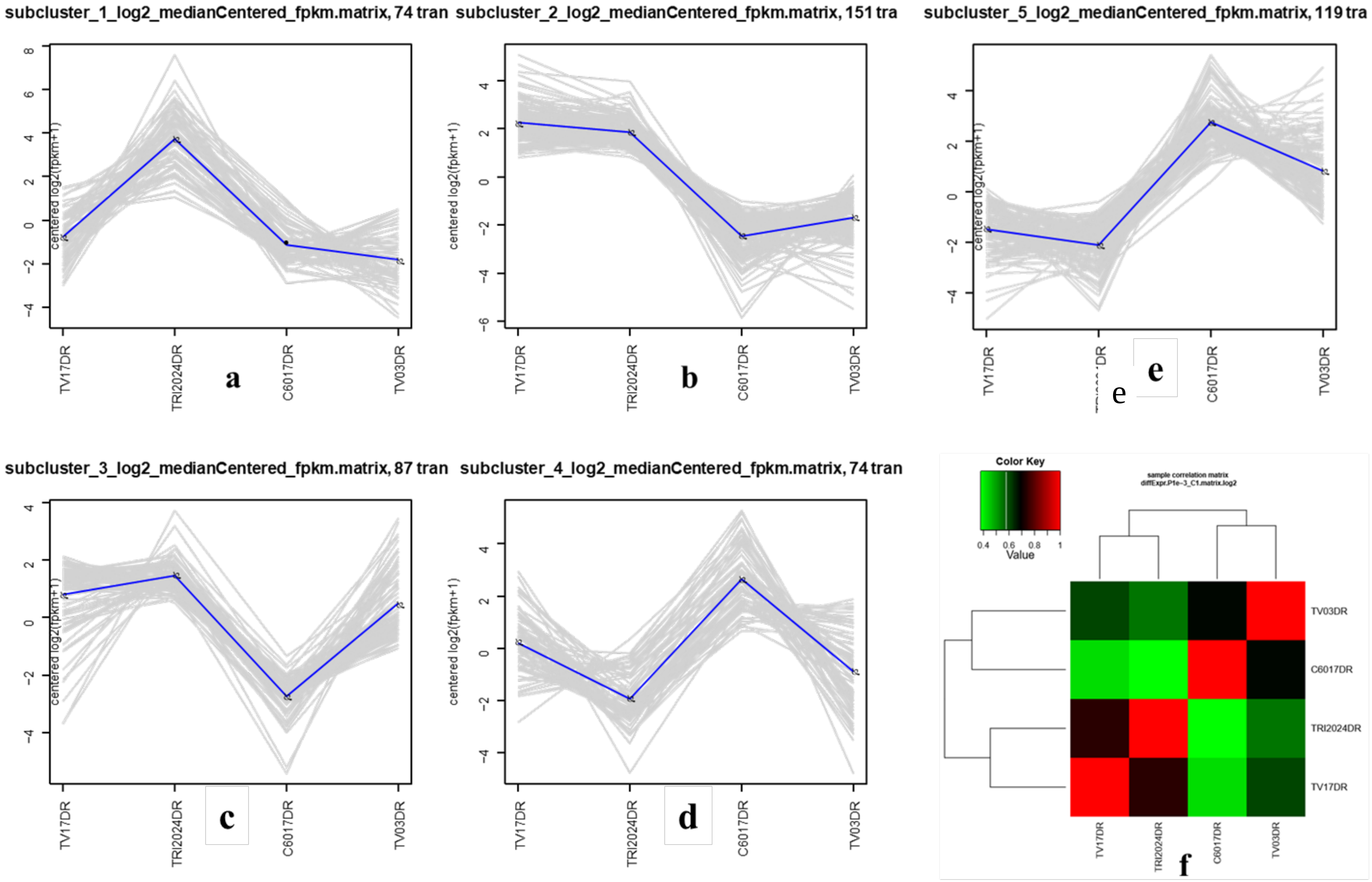

subcluster_1_log2_medianCentered_fpkm.matrix, 74 tran
subcluster_2_log2_medianCentered_fpkm.matrix, 151 tra
subcluster_5_log2_medianCentered_fpkm.matrix, 119 tra
subcluster_3_log2_medianCentered_fpkm.matrix, 87 tran
subcluster_4_log2_medianCentered_fpkm.matrix, 74 tran
centered log2(fpkm+1)
TV17DR
TRI2024DR
C6017DR
TV03DR
a
b
c
d
e
f
Color Key
Value
0.4
0.6
0.8
1
sample correlation matrix
diffExpr.P1e-3_C1.matrix.log2

Figure S4a (Molecular Function)

DT_G1

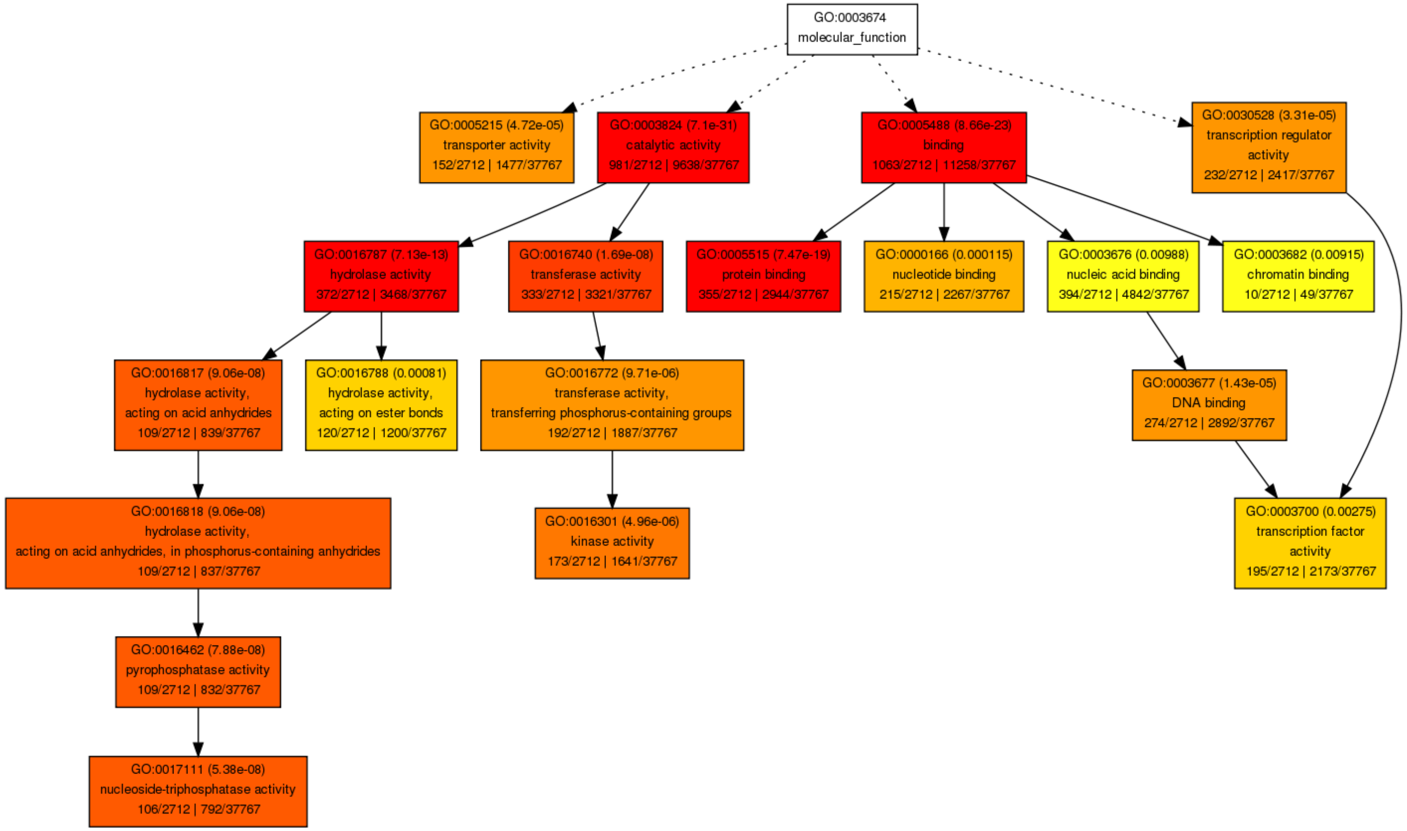

Figure S4b (Biological Function)

DT_G1

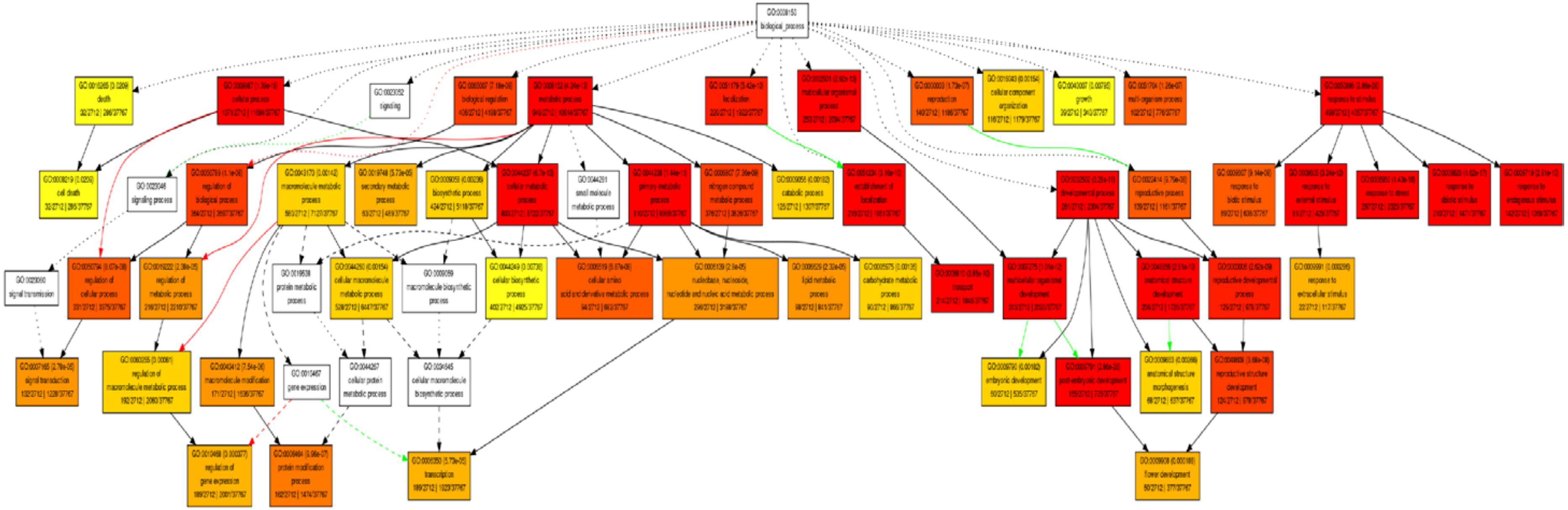

Figure S4c (Cellular Component)

DT_G1

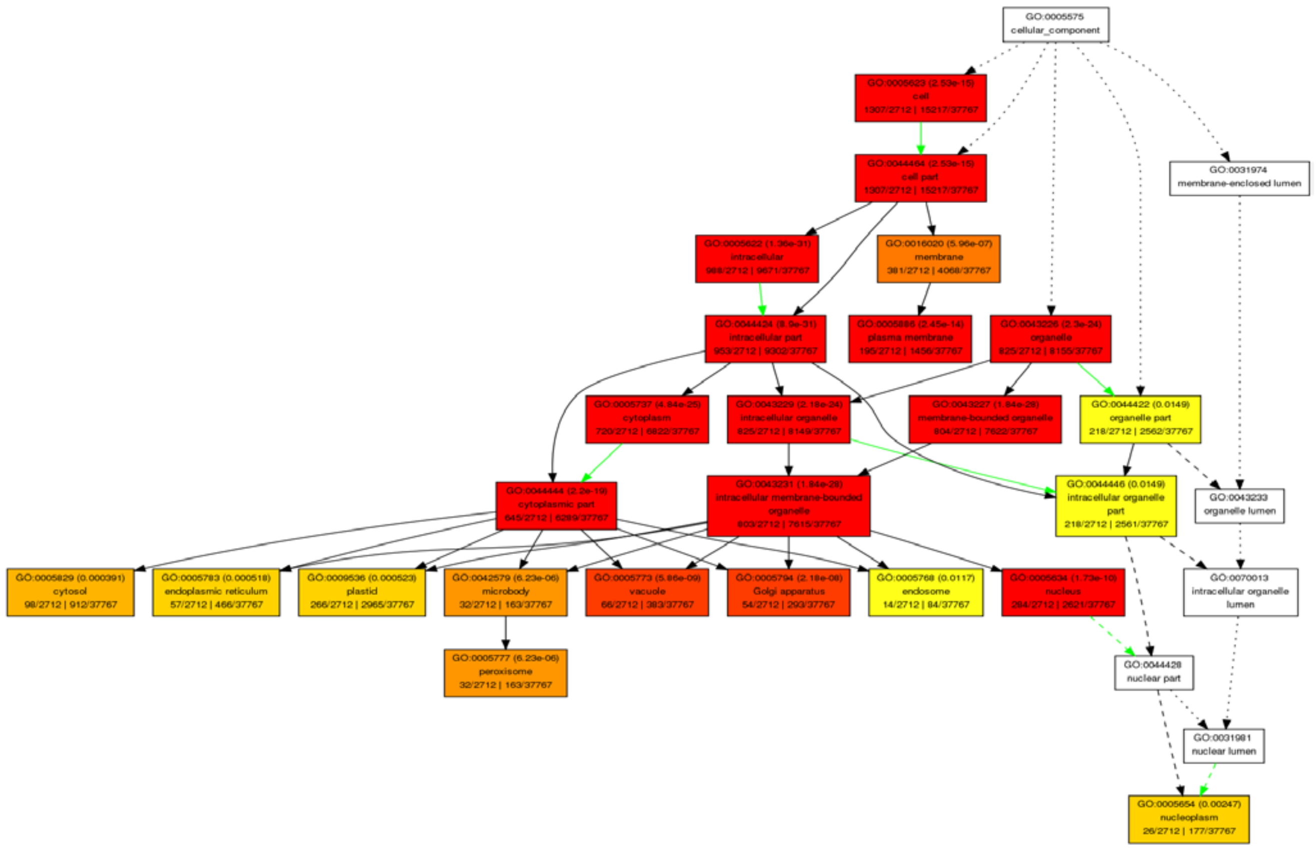

Figure S5a (Molecular Function)

DT_G2

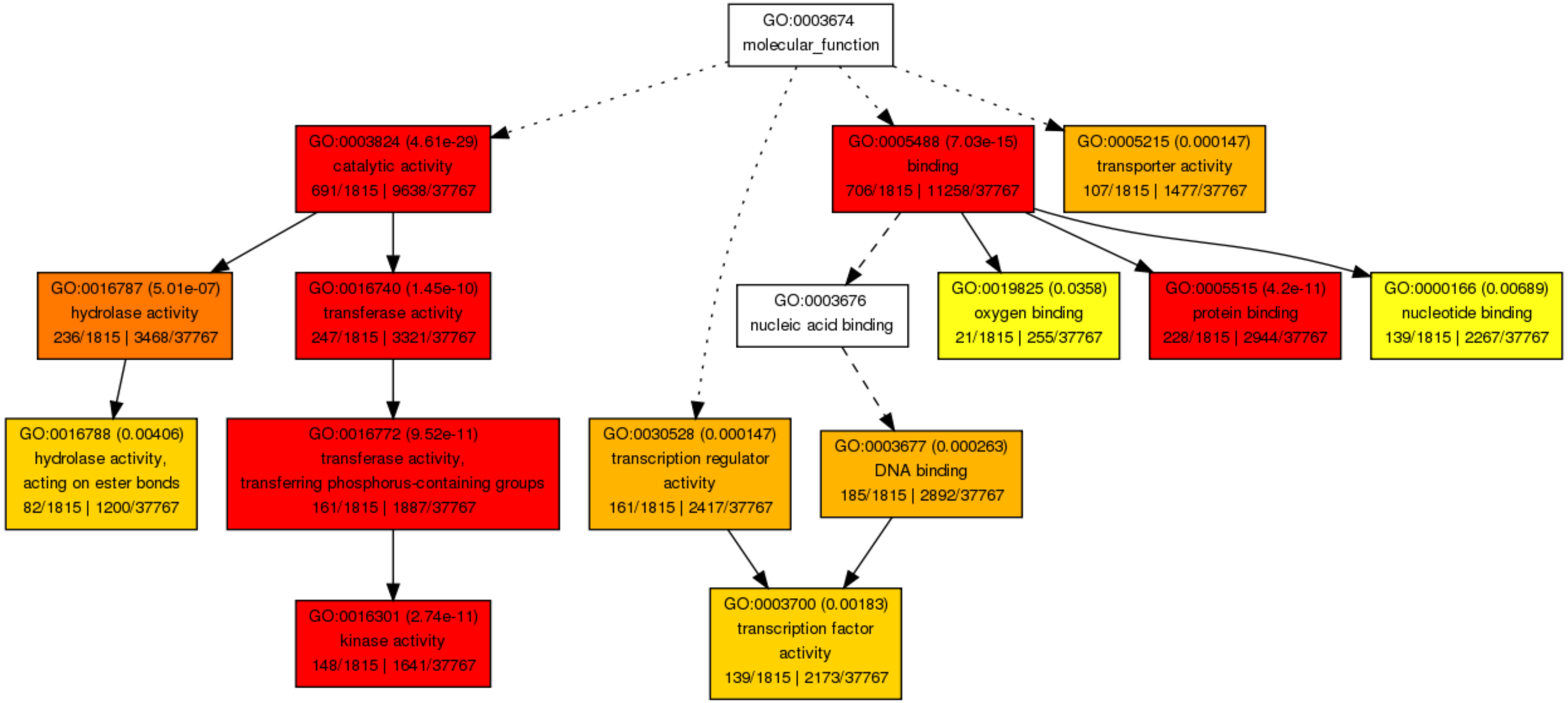

Figure S5b (Biological Function)

DT_G2

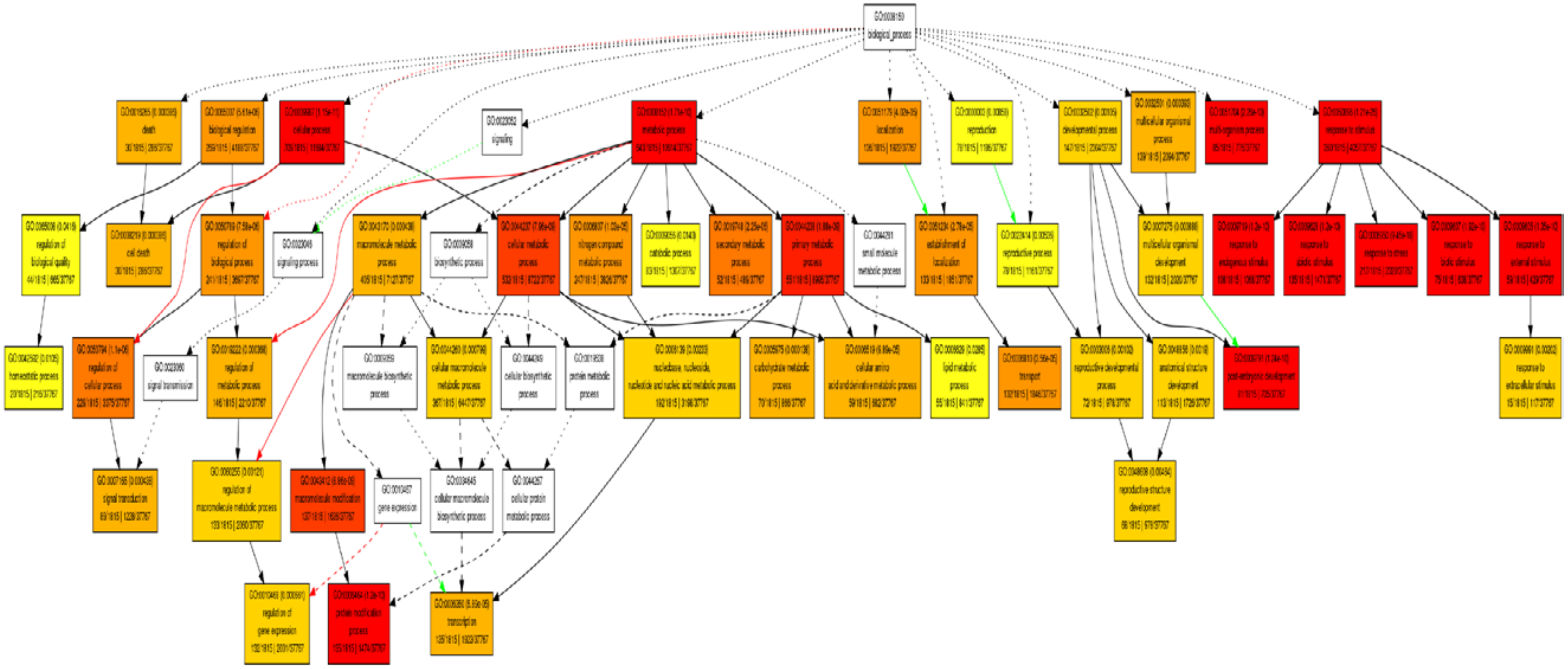

Figure S5c (Cellular Component)

DT_G2

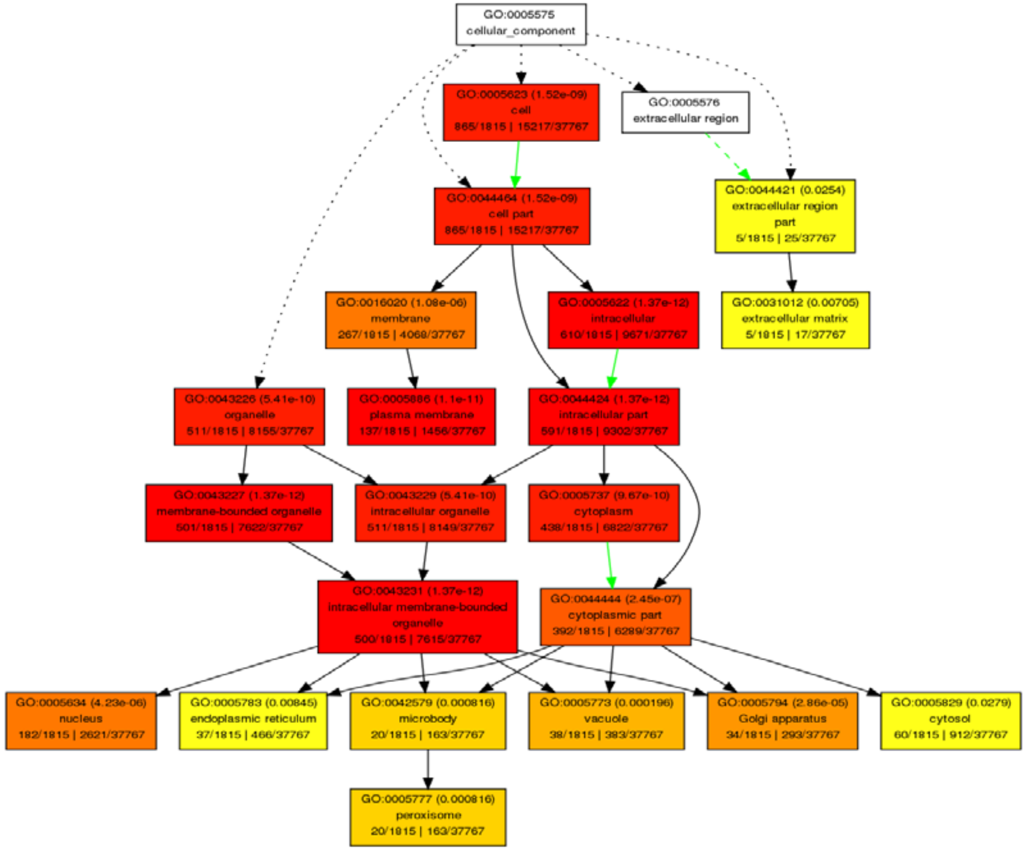

Figure S6a (Molecular Function)

DS_G1

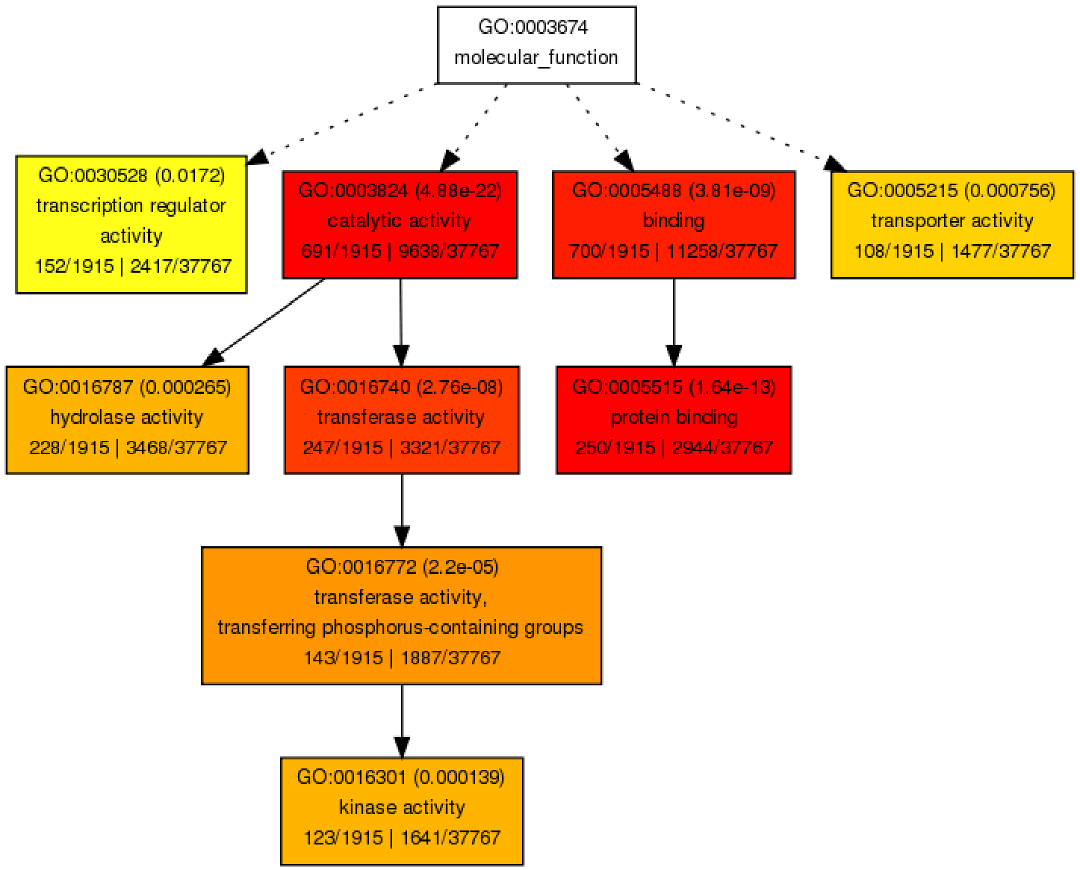

Figure S6b (Biological Function)

DS_G1

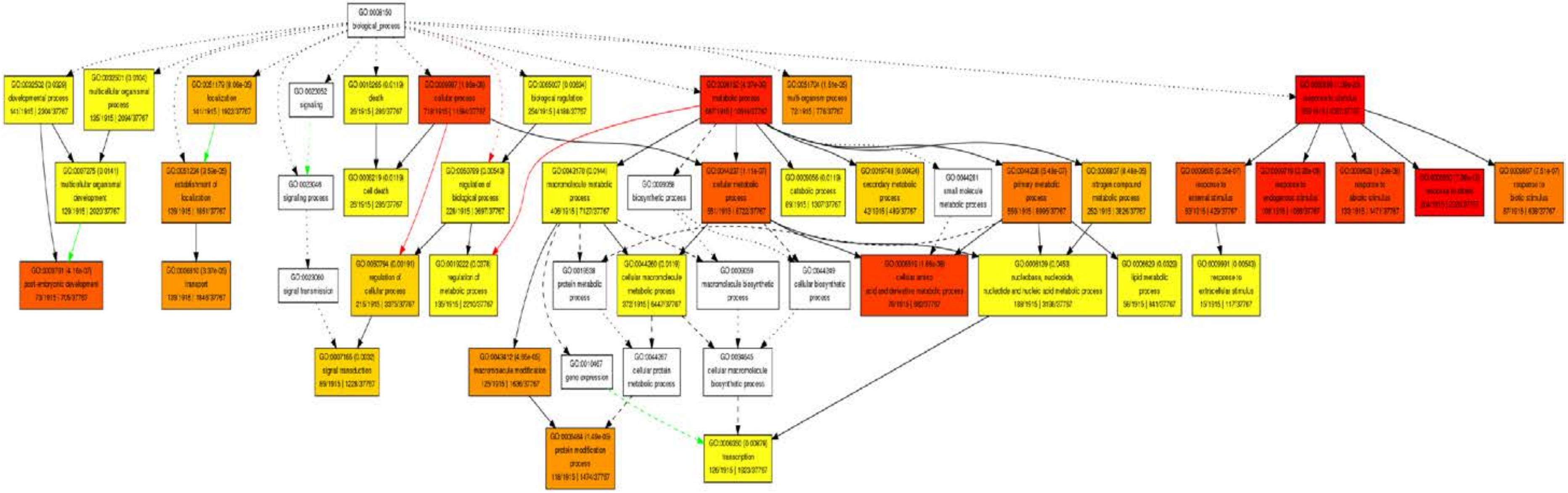

Figure S6c (Cellular Component)

DS_G1

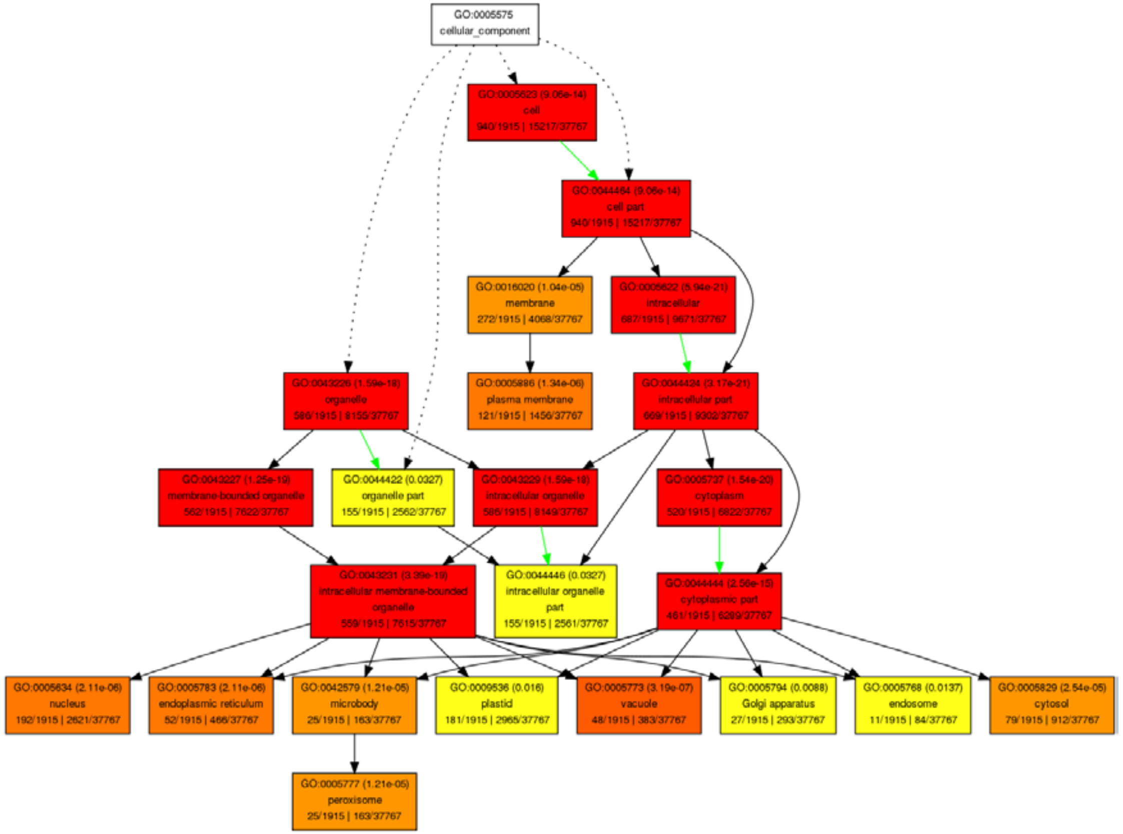

Figure S7a (Molecular Function)

DS_G2

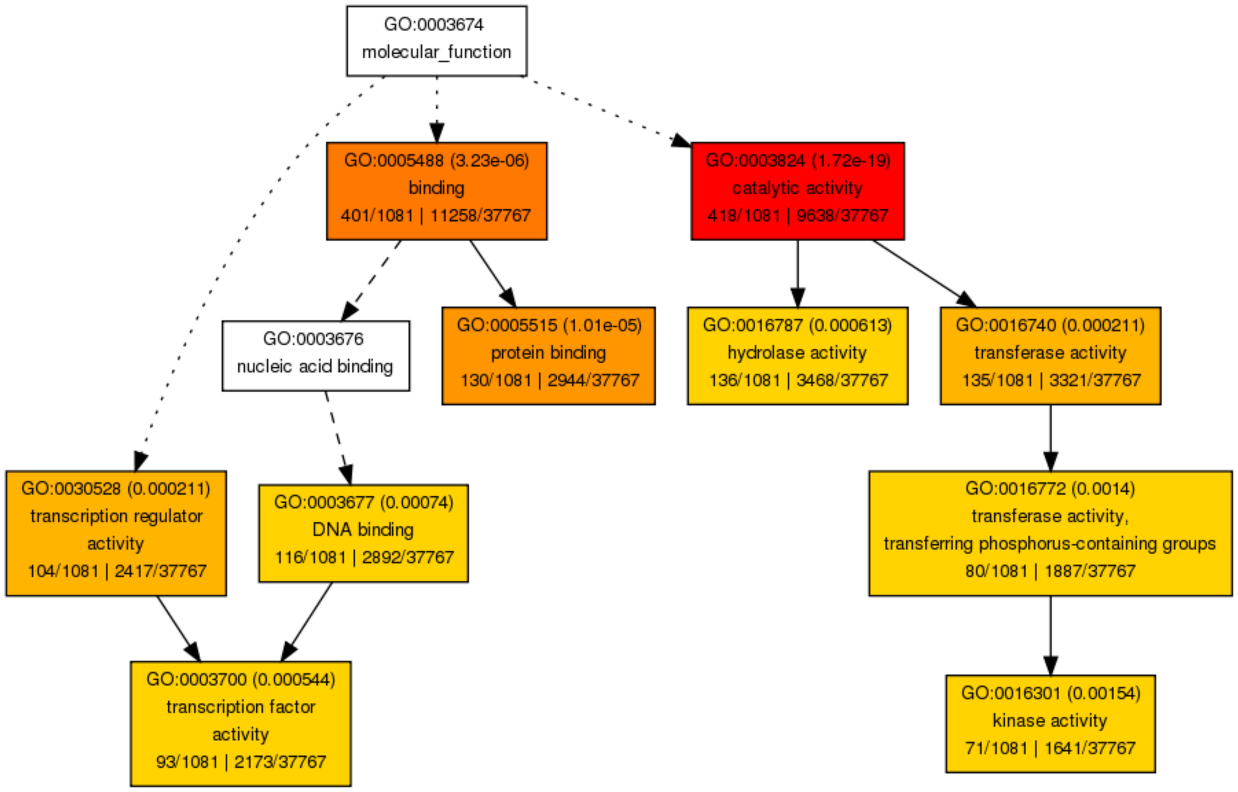

Figure S7b (Biological Function)

DS_G2

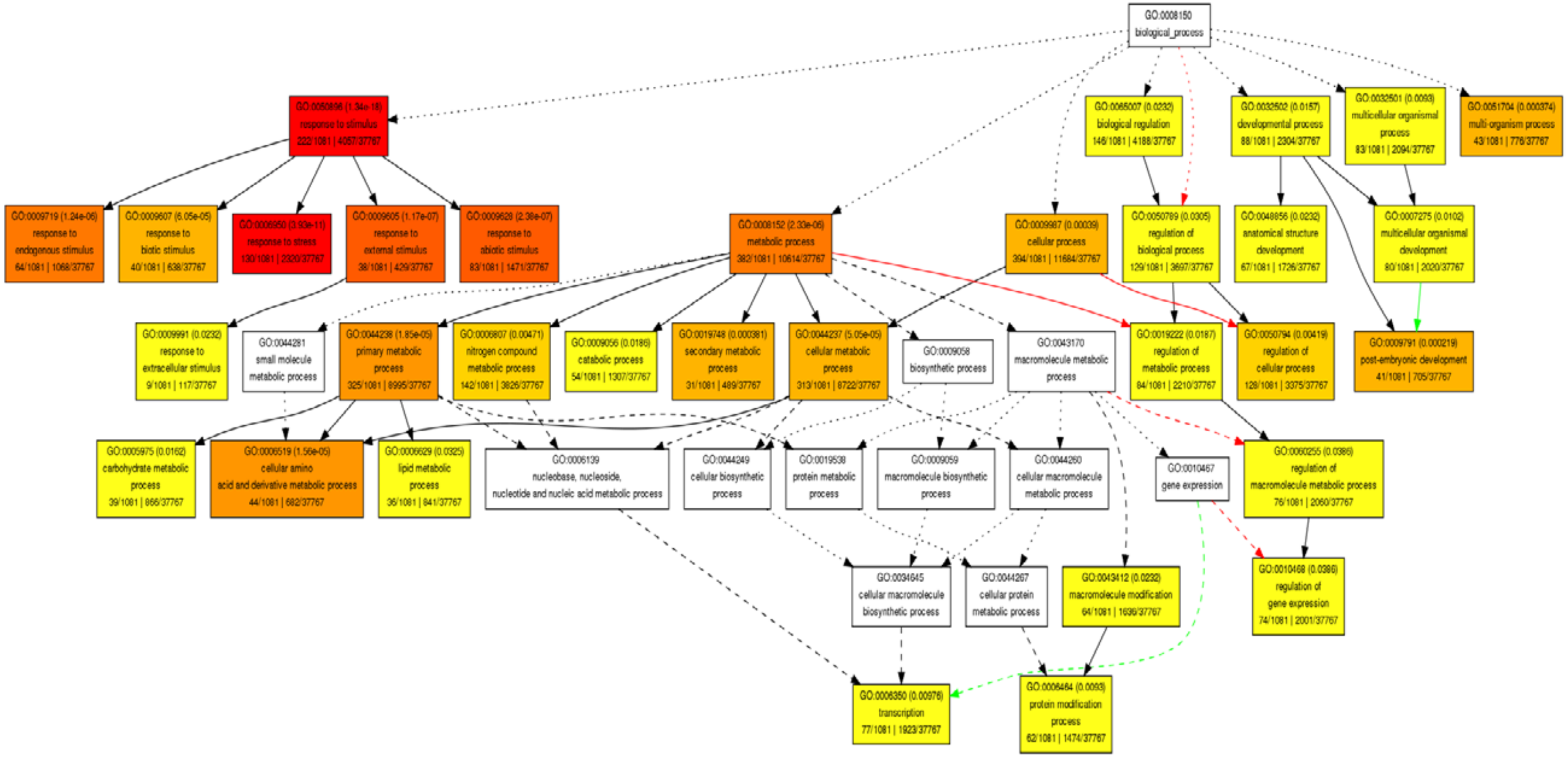

Figure S7a (Cellular Component)

DS_G2

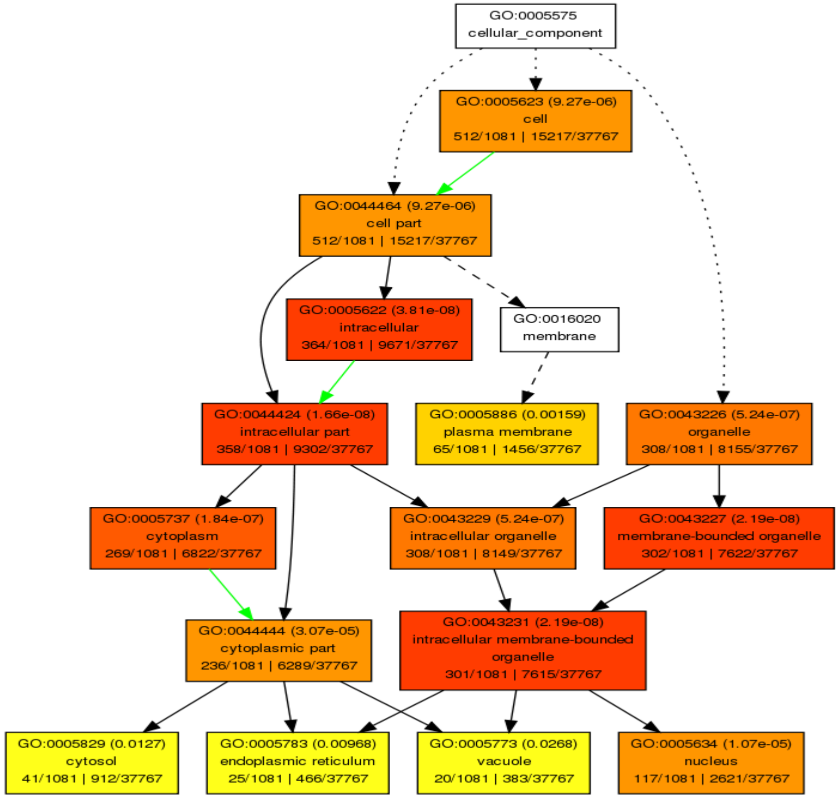

Figure S8

DT_G1_T and DT_G2_T

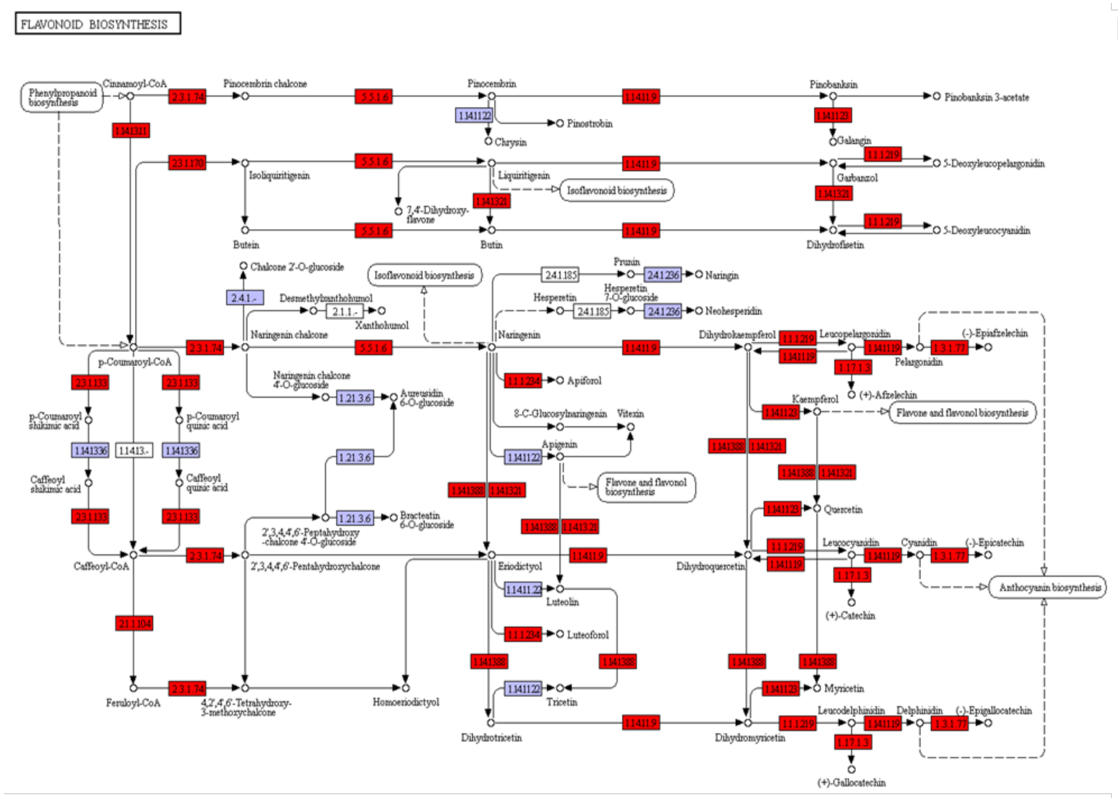

DS_G1_T and DS_G2_T

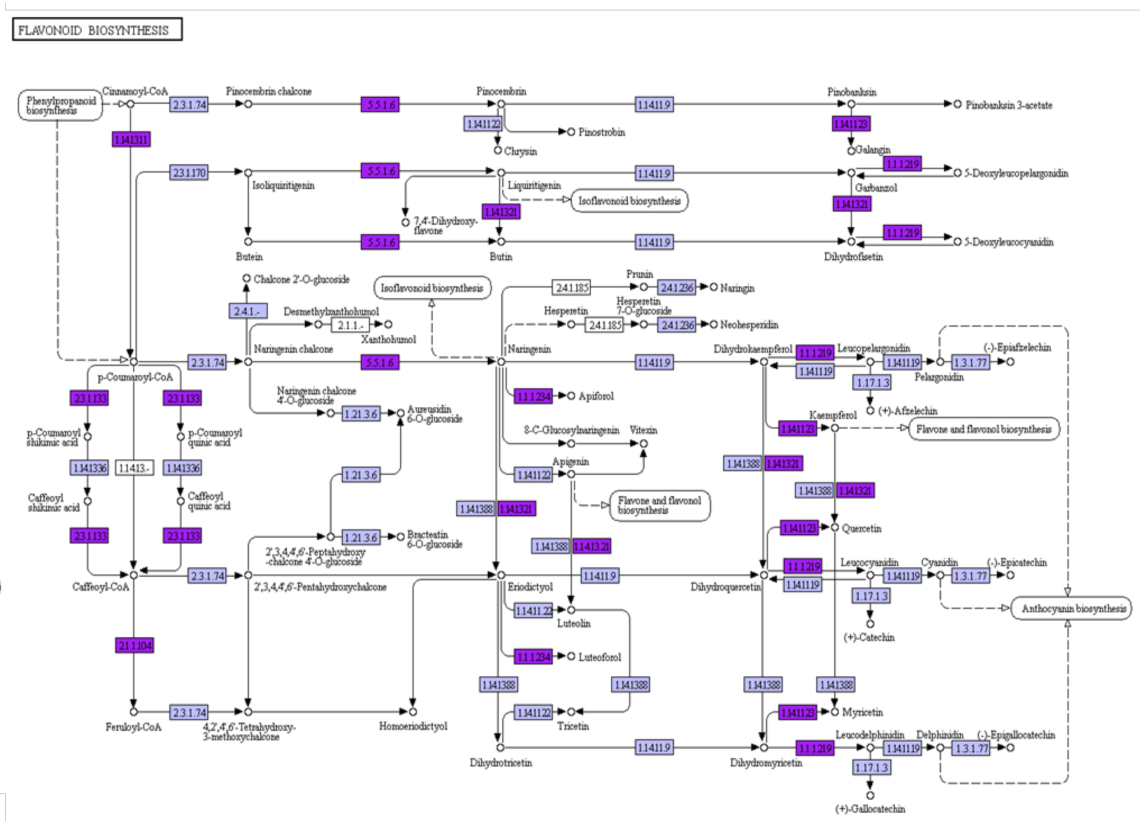

Figure S9

DT_G1_T and DT_G2_T

DS_G1_T and DS_G2_T

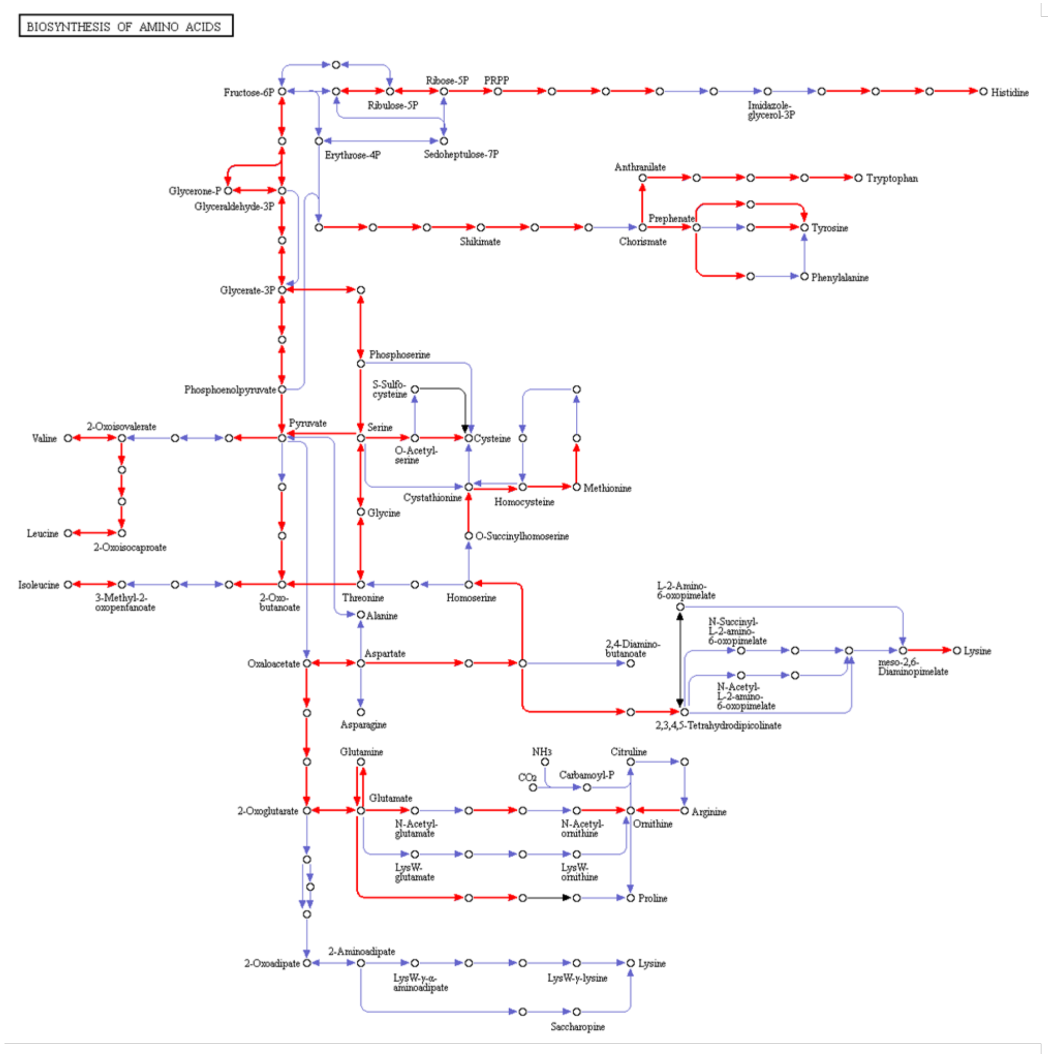

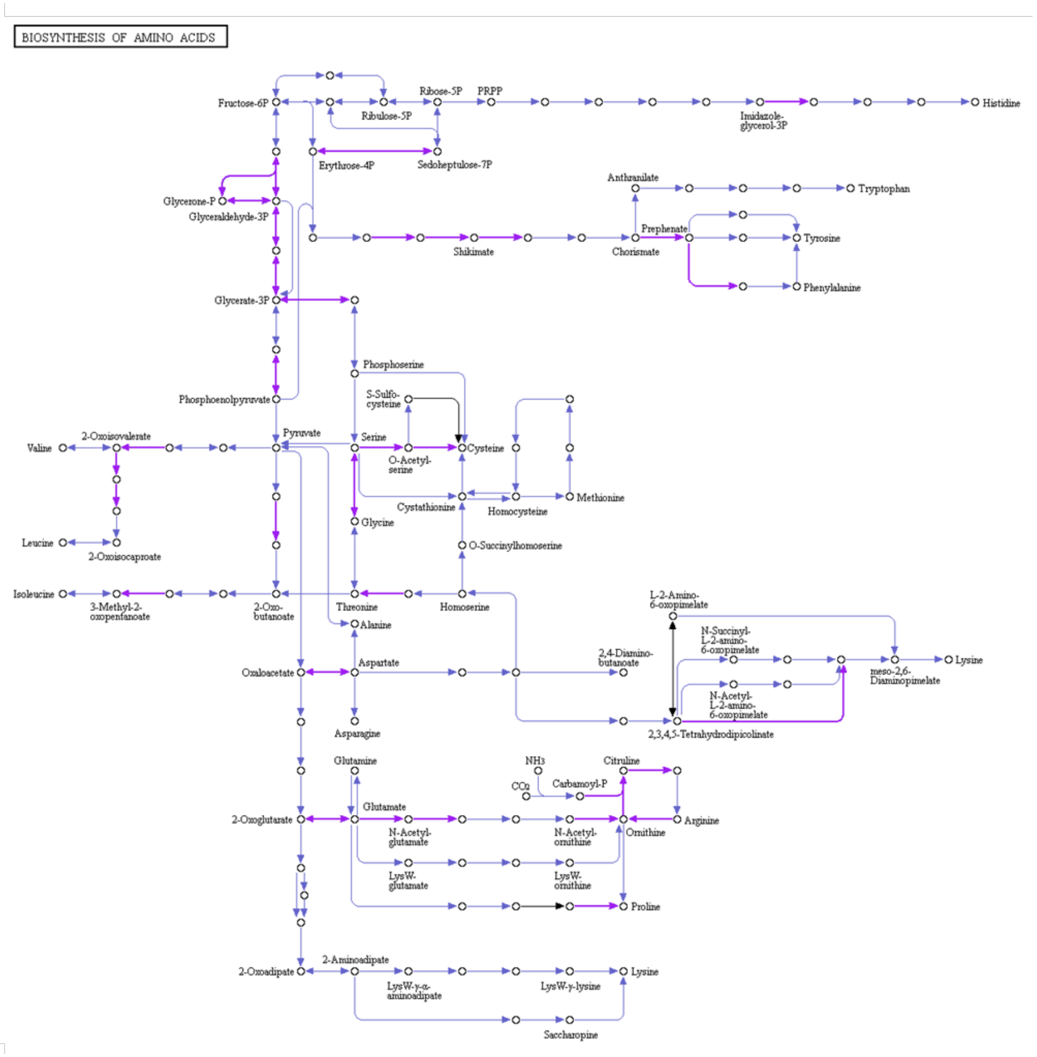

Figure S10

DT_G1_T and DT_G2_T

DS_G1_T and DS_G2_T

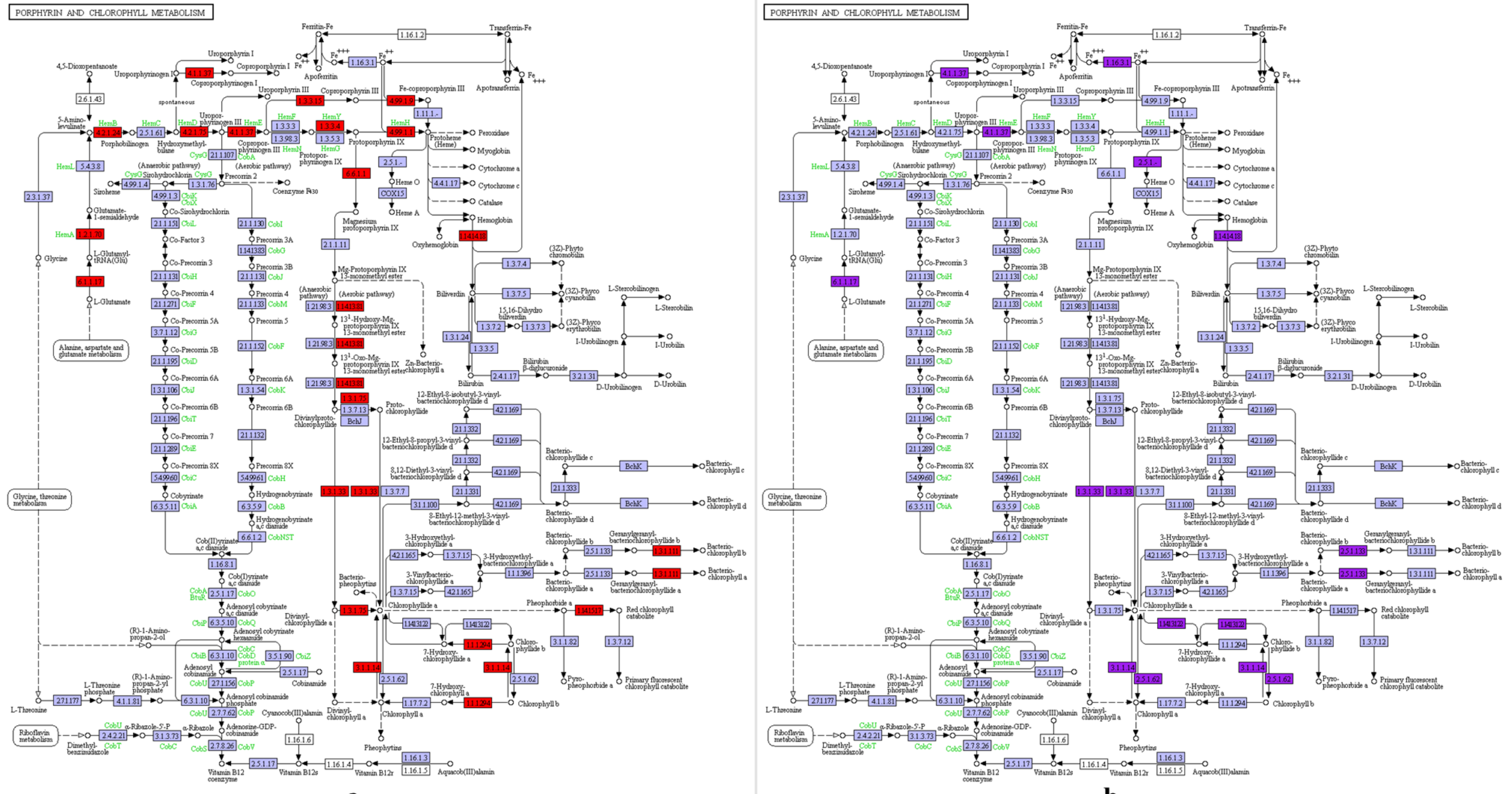

a

b

Figure S11

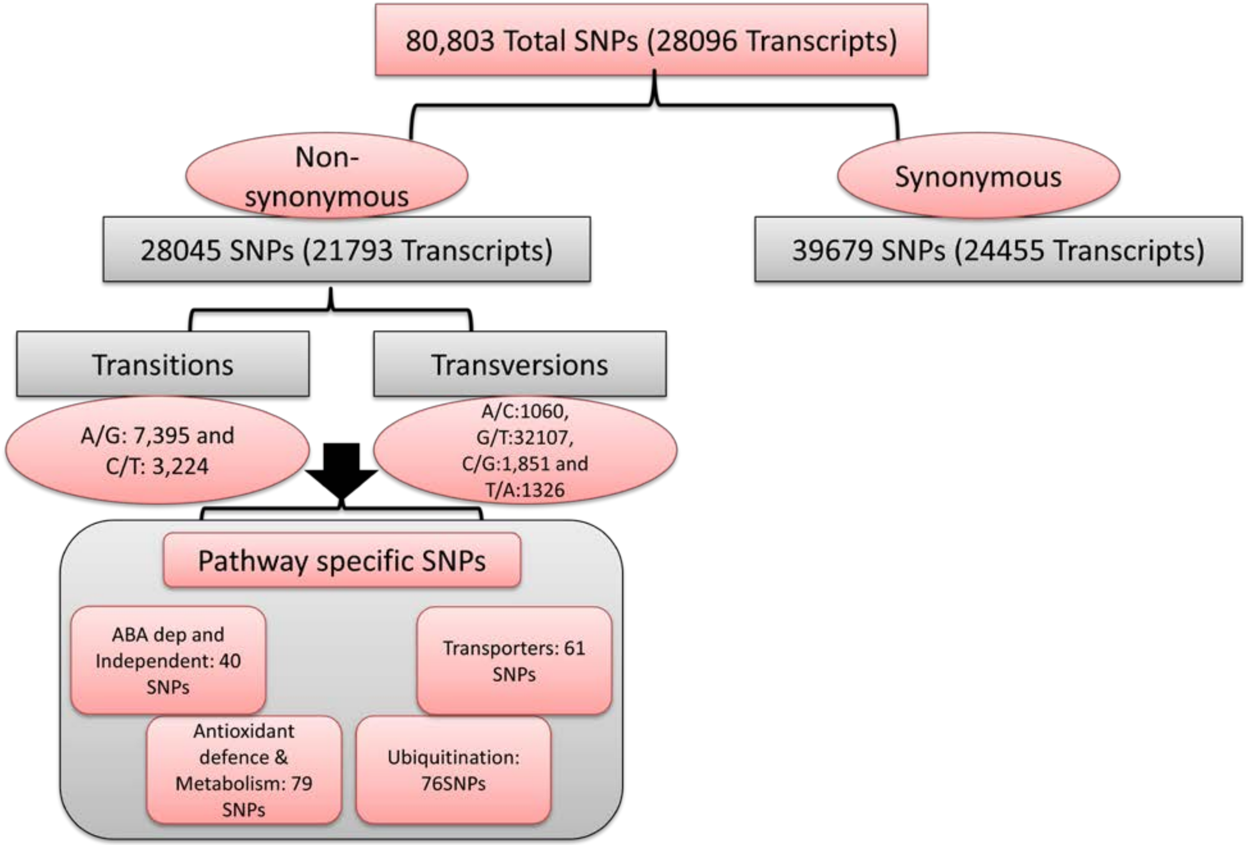
80,803 Total SNPs (28096 Transcripts)
Non-synonymous
Synonymous
28045 SNPs (21793 Transcripts)
39679 SNPs (24455 Transcripts)
Transitions
Transversions
A/G: 7,395 and C/T: 3,224
A/C:1060, G/T:32107, C/G:1,851 and T/A:1326
Pathway specific SNPs
ABA dep and Independent: 40 SNPs
Transporters: 61 SNPs
Antioxidant defence & Metabolism: 79 SNPs
Ubiquitination: 76SNPs

Figure S12

Biological Process

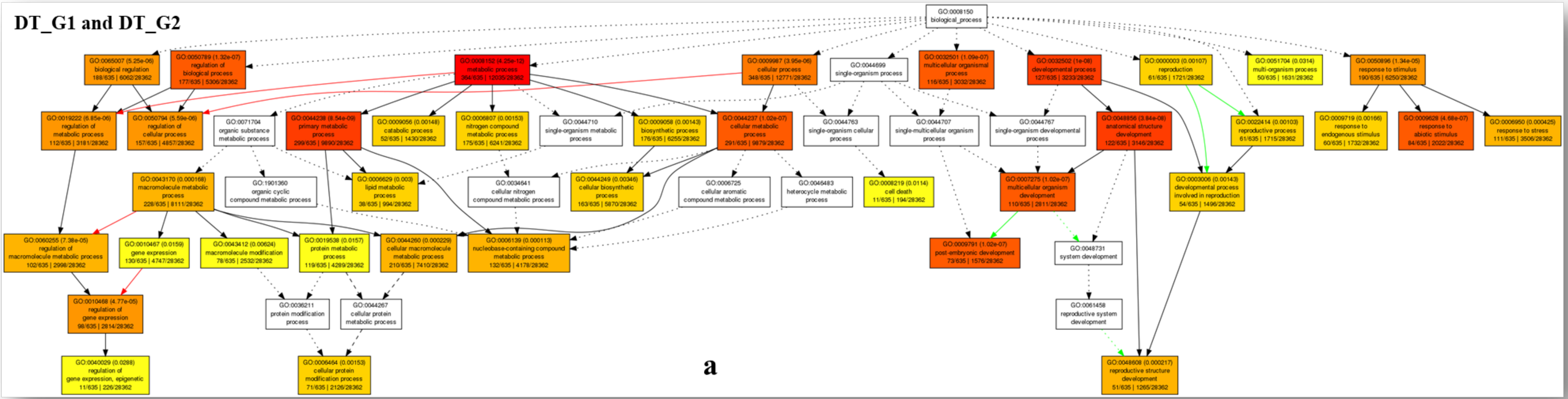

a

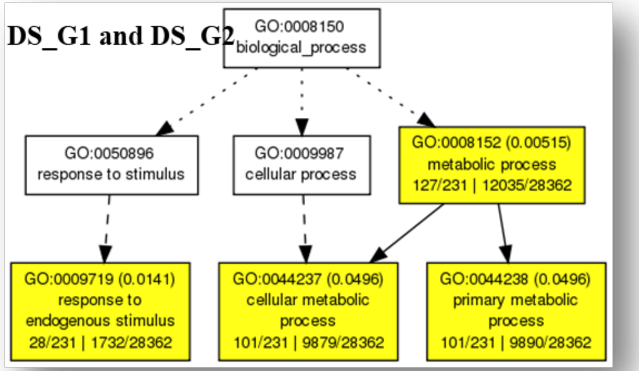

b

Figure S13

Cellular Component

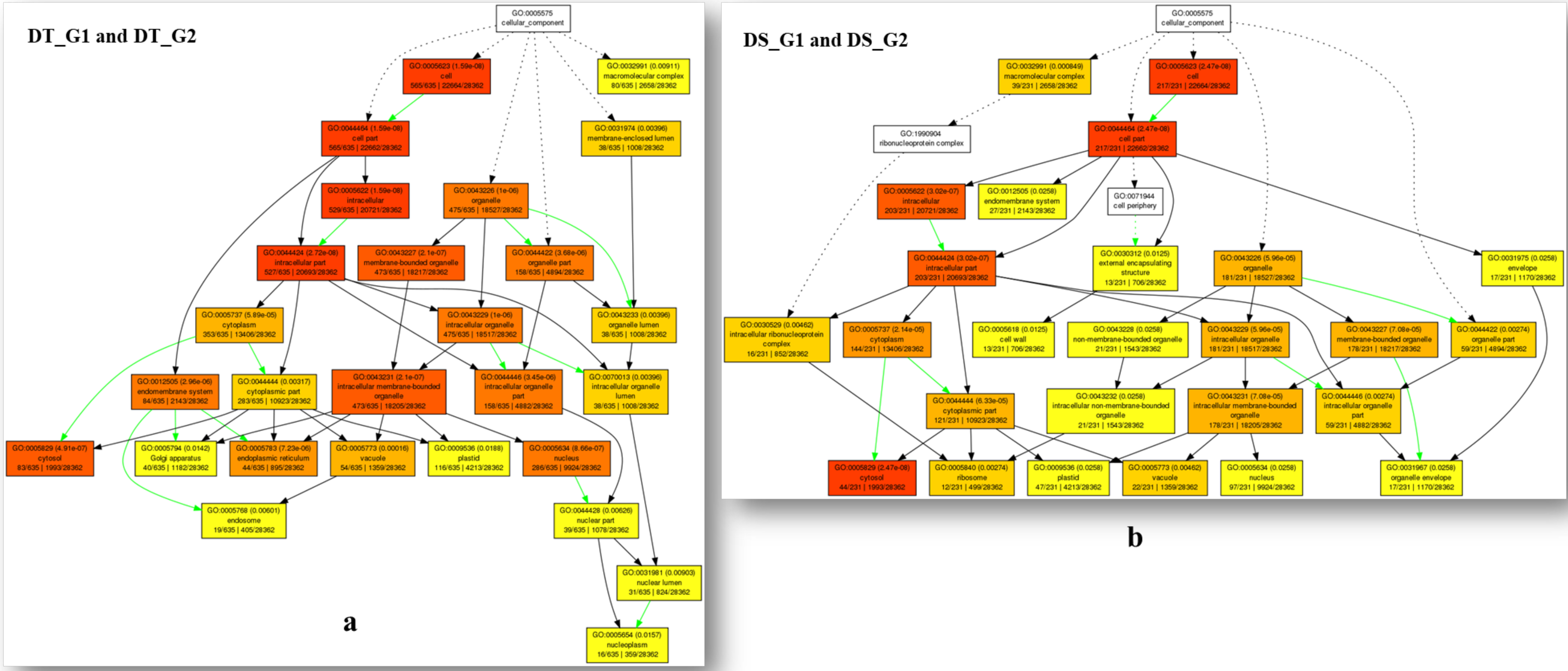

Figure S14

Molecular Function

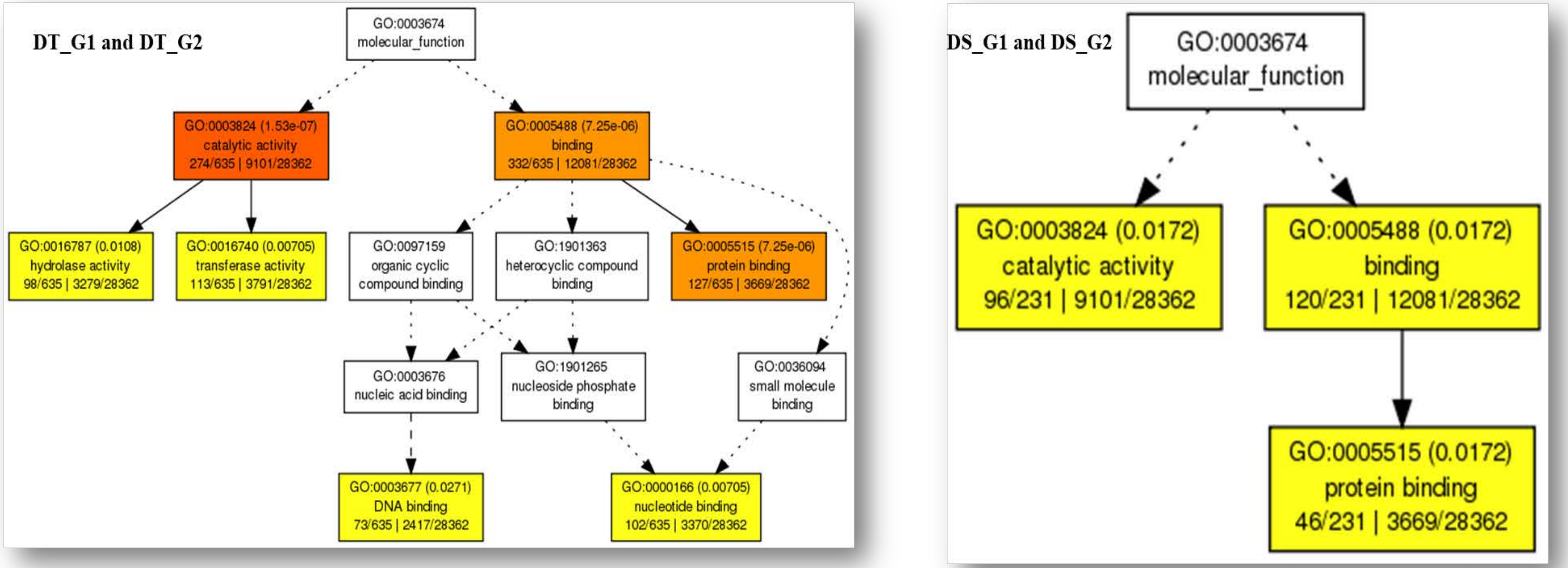

a

b

Figure S15

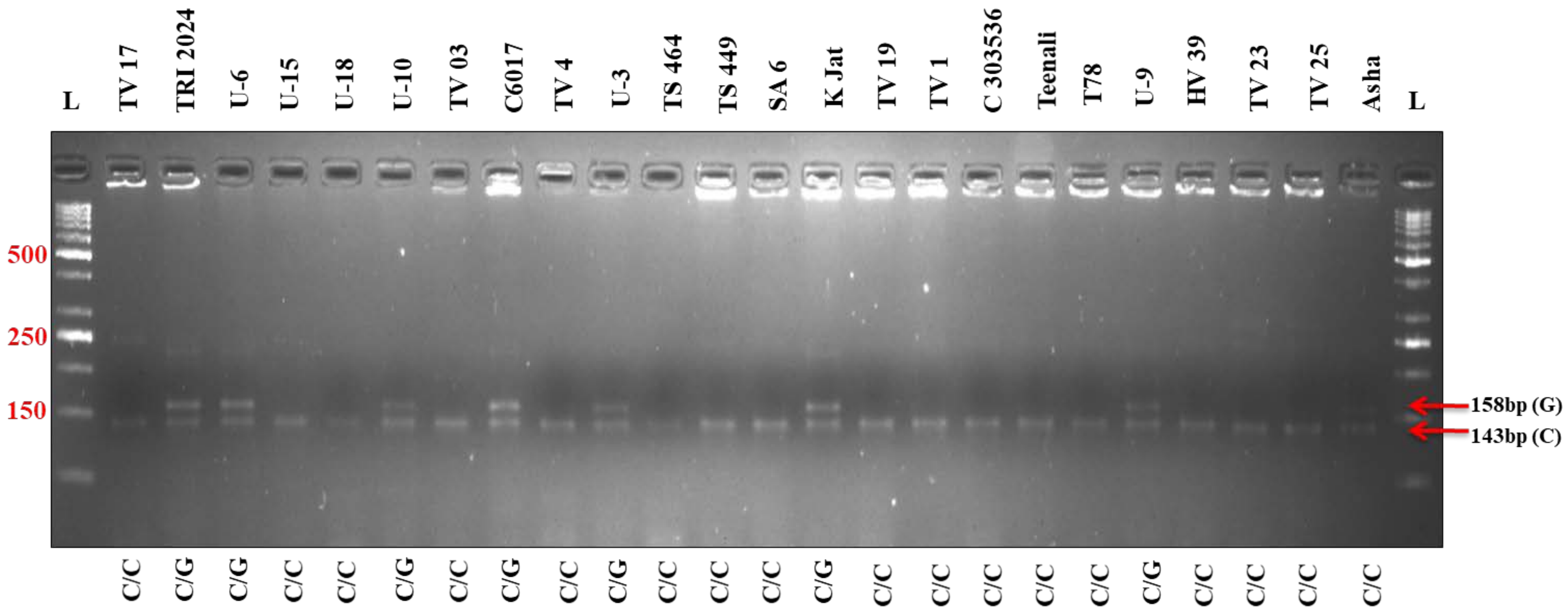

L
TV 17
TRI 2024
U-6
U-15
U-18
U-10
TV 03
C6017
TV 4
U-3
TS 464
TS 449
SA 6
K Jat
TV 19
TV 1
C 303536
Teenali
T78
U-9
HV 39
TV 23
TV 25
Asha
L
500
250
150
158bp (G)
143bp (C)
C/C
C/G
C/G
C/C
C/C
C/G
C/C
C/G
C/C
C/G
C/C
C/C
C/C
C/G
C/C
C/C
C/C
C/C
C/C
C/G
C/C
C/C
C/C
C/C

Figure S15

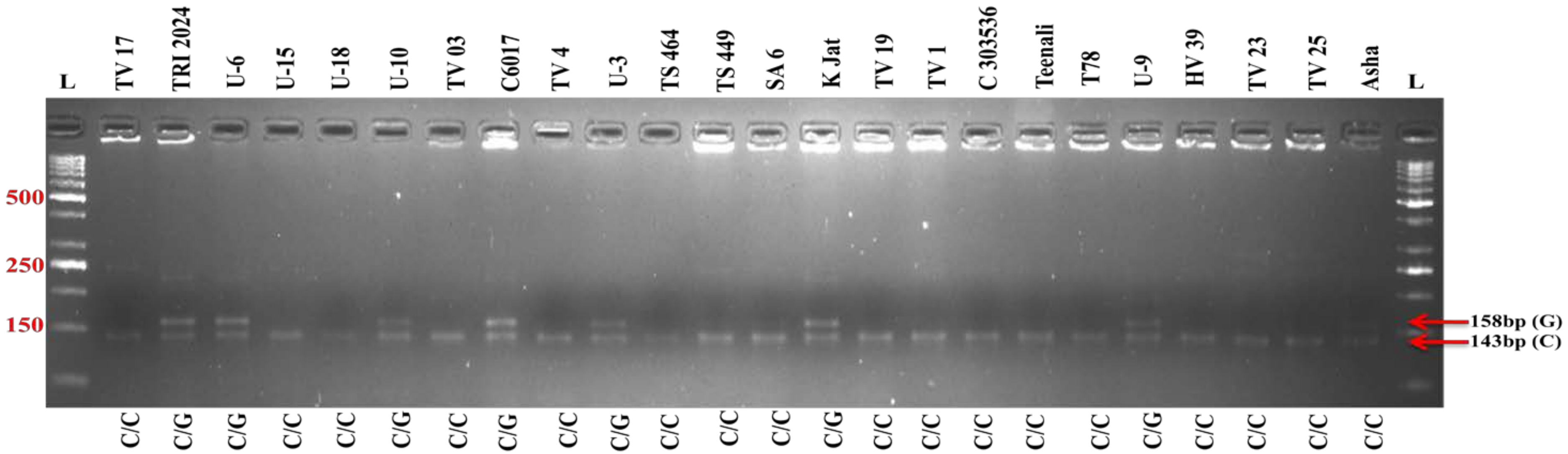
L
TV 17
TRI 2024
U-6
U-15
U-18
U-10
TV 03
C6017
TV 4
U-3
TS 464
TS 449
SA 6
K Jat
TV 19
TV 1
C 303536
Teenali
T78
U-9
HV 39
TV 23
TV 25
Asha
L
500
250
150
158bp (G)
143bp (C)
C/C
C/G
C/G
C/C
C/C
C/G
C/C
C/G
C/C
C/G
C/C
C/C
C/C
C/G
C/C
C/C
C/C
C/C
C/C
C/G
C/C
C/C
C/C
C/C

Table S1: **Summary of *de novo* assembly of Tea transcriptome.**

**Quality Filtering**	
**Raw Reads**	140205740
**Filtered Reads**	123602034
**TRINITY Assembly Statistics**	
**No. of assembled transcripts**	67,093
**Minimum sequence length**	301
**Maximum sequence length**	17,990
**N50 length**	1501
**Average sequence length**	1086
**Clustering**	
**No. of transcripts**	54,508
**No. of genes**	35,379
**No. of Isoforms**	14,077

Table S2: Summary of functional annotation of transcripts with public databases.

Databases	Transcripts Annotated	%Annotation
nr Blast	52,484	78.20%
Swiss-Prot	41,475	61.08%
TFIIdb	27,204	40.54%
TAIR 10	47,962	71.40%
KEGG	13,454	20.05%

Table S9: Detail of genotypes used for SNP validation using Tetra ARM PCR.

S. No.	Genotypes	Varietal Type
**1**	TV 17	Assam Hybrid
**2**	TRI 2024	Assam
**3**	U 6	Assam
**4**	U 15	China
**5**	U 18	Cambod
**6**	U 10	China
**7**	TV 03	Assam
**8**	C 6017	Cambod
**9**	TV 04	Assam
**10**	U 3	Assam
**11**	TS 464	Assam Hybrid
**12**	TS 449	Assam Hybrid
**13**	SA 6	Assam
**14**	K Jat	China Hybrid
**15**	TV 19	Cambod
**16**	TV 1	Assam China Hybrid
**17**	C 303536	China
**18**	Teenali	Assam
**19**	T 78	China
**20**	U 9	Assam
**21**	HV 39	China
**22**	TV 23	Cambod
**23**	TV 25	Cambod
**24**	K Asha	China Hybrid

Supplementary Legends

Supplementary figures

Figure S1: (a) Overall GO annotation of transcripts categorized into Cellular Component, Biological Processes and Molecular Functions. (b) Tree map representation of transcripts annotated in KEGG Pathway: Area of box represents the number of transcripts mapped to the pathway. (c) Plant TF represented in the form of scattered plot.

Figure S2: Venn diagram representing overall DE transcripts in *de novo* assembly

Figure S3a: (a-g) Clustering of pair-wise DE transcripts in Control and Treatments (FC ≥ 2; p-value $< 1e-4$), (h) Correlation plot between Control and Treatments.

Figure S3c: (a-e) Clustering of pair-wise DE transcripts in Tolerant and Sensitive (FC ≥ 2; p-value $< 1e-4$), (f) Correlation plot between Tolerant and Sensitive.

Figure S4: GO enrichment analysis of up-regulated transcripts in DT_G1. (a) Molecular Function; (b) Biological processes (C) Cellular component.

Figure S5: GO enrichment analysis of up-regulated transcripts in DT_G2. (a) Molecular Function; (b) Biological processes (C) Cellular component.

Figure S6: GO enrichment analysis of up-regulated transcripts in DS_G1. (a) Molecular Function; (b) Biological processes (C) Cellular component.

Figure S7: GO enrichment analysis of up-regulated transcripts in DS_G2. (a) Molecular Function; (b) Biological processes (C) Cellular component.

Figure S8: KEGG enrichment analysis[56] of up-regulated transcripts in Flavonoid pathway in (a) Tolerant genotypes (DT_G1_T & DT_G2_T) and (b) Sensitive genotypes (DS_G1_T & DS_G2_T) (www.kegg.jp/kegg/kegg1.html).

Figure S9: KEGG enrichment analysis[56] of up-regulated transcripts in Biosynthesis of amino acids in (a) Tolerant genotypes (DT_G1_T & DT_G2_T) and (b) Sensitive genotypes (DS_G1_T & DS_G2_T) (www.kegg.jp/kegg/kegg1.html).

Figure S10: KEGG enrichment analysis[56] of up-regulated transcripts in porphyrin and chlorophyll metabolism in (a) Tolerant genotypes (DT_G1_T & DT_G2_T) and (b) Sensitive genotypes (DS_G1_T & DS_G2_T) (www.kegg.jp/kegg/kegg1.html).

Figure S11: Flowchart of total SNPs identified from the transcriptome data and

Figure S12: GO enrichment of transcripts containing SNPs; Biological processes (a) Tolerant genotypes (DT_G1_T & DT_G2_T) and (b) Sensitive genotypes (DS_G1_T & DS_G2_T)

Figure S13: GO enrichment of transcripts containing SNPs; Cellular Component (a) Tolerant genotypes (DT_G1_T & DT_G2_T) and (b) Sensitive genotypes (DS_G1_T & DS_G2_T)

Figure S14: GO enrichment of transcripts containing SNPs; Molecular Functions (a) Tolerant genotypes (DT_G1_T & DT_G2_T) and (b) Sensitive genotypes (DS_G1_T & DS_G2_T)

Figure S15: Validation of SNPs using tetra primer ARM-PCR.

Supplementary Tables

Supplementary Table S1: Summary of *de novo* assembly of Tea transcriptome.

Supplementary Table S2: Summary of functional annotation of transcripts with public databases.

Supplementary Table S3: Functional annotation of assembled transcripts with multifarious databases and tea reference genome localisation of transcripts; GO annotation; KEGG annotation.

Supplementary Table S4: Differential Gene Expression extracted from *de novo* assembly using EdgeR tools. DGE between C 6017 vs TRI 2024; TRI 2024 vs TV 03; C 6017 vs TV 17; TV 03 vs TV 17.

Supplementary Table S5: Tea reference genome based differential gene expression using tuxedo pipeline.

Supplementary Table S6a: Shortlisted differentially expressed transcripts of targeted pathways in response to drought stress among Control and Treated plants.

Supplementary Table S6b: Shortlisted differentially expressed transcripts of targeted pathways in response to drought stress among Tolerant and Sensitive genotypes.

Supplementary Table S7: List of primer sequences of selected transcripts used for quantitative reverse transcriptase-polymerase chain reaction (qRT-PCR) analysis.

Supplementary Table S8: Non-synonymous SNPs identification in CDS region of denovo assembled transcripts.

Supplementary Table S9: Detail of genotypes used for SNP validation using Tetra ARM PCR**.**

Supplementary Table S10: Synthesized and validated 37 SNPs, identified in drought stress responsive transcripts.
